# Parameter estimation and determinability analysis applied to *Drosophila *gap gene circuits

**DOI:** 10.1186/1752-0509-2-83

**Published:** 2008-09-25

**Authors:** Maksat Ashyraliyev, Johannes Jaeger, Joke G Blom

**Affiliations:** 1 CWI, Kruislaan 413, 1098 SJ Amsterdam, The Netherlands; 2 Laboratory for Development & Evolution, University Museum of Zoology, Department of Zoology, University of Cambridge, Cambridge CB2 3EJ, UK

## Abstract

**Background:**

Mathematical modeling of real-life processes often requires the estimation of unknown parameters. Once the parameters are found by means of optimization, it is important to assess the quality of the parameter estimates, especially if parameter values are used to draw biological conclusions from the model.

**Results:**

In this paper we describe how the quality of parameter estimates can be analyzed. We apply our methodology to assess parameter determinability for gene circuit models of the gap gene network in early *Drosophila* embryos.

**Conclusion:**

Our analysis shows that none of the parameters of the considered model can be determined individually with reasonable accuracy due to correlations between parameters. Therefore, the model cannot be used as a tool to infer quantitative regulatory weights. On the other hand, our results show that it is still possible to draw reliable qualitative conclusions on the regulatory topology of the gene network. Moreover, it improves previous analyses of the same model by allowing us to identify those interactions for which qualitative conclusions are reliable, and those for which they are ambiguous.

## Background

Many real-life processes can be modeled by non-linear Ordinary Differential Equations (ODEs) or Partial Differential Equations (PDEs). In developmental biology, for instance, systems of reaction-diffusion equations are used to model spatio-temporal patterns of gene expression [1]. A common difficulty is that the model equations usually have a large number of unknown parameters, such as weights for regulatory interactions, diffusion coefficients, decay and reaction rates, etc. Sometimes, it is feasible to determine the missing parameters experimentally, but in most cases this is difficult or even impossible. However, one can usually measure other quantities involved in the model. For instance, experimentalists can quantify mRNA or protein concentrations using microarrays, quantitative PCR, in situ hybridization or immunofluorescence. Unknown model parameters can then be found by parameter estimation techniques based on fitting the model solution to the measured gene expression data.

Whether the parameters for the mathematical model can be found assuming that sufficient and error-free data is available is the subject of *a priori *or *structural identifiability *analysis. Once the parameter estimates have been computed, it is very important to know how reliable they are. An a *posteriori *or *practical identifiability *study can show how well the parameters have been determined given a data set that is possibly sparse and noisy. For the subject of structural and practical identifiability we refer to [2-4] and references therein. Ideally, one would wish to determine all parameters accurately enough. In practice, however, this is usually not possible and one has to face an uncertainty in the parameter values. This can be due to several reasons: First, the model could be 'wrong'. In this paper, we do not focus on this possibility assuming that the 'right' model is available (i.e. a model which represents the underlying mechanism of the modeled process accurately and correctly). Second, the data used for fitting could be insufficient or too noisy. Finally, a recent study by Gutenkunst et al. [5] revealed that even if a correct model is used with a comprehensive set of data, many models used in systems biology still exhibit parameter 'sloppiness'. This means that some model parameters can be determined with great certainty ('stiff' parameters), while estimates of other ('sloppy') parameters can vary by orders of magnitude without significantly influencing the quality of the fit. Parameter sloppiness implies that very different sets of estimated parameters can lead to accurate model predictions. Therefore, it is not a serious problem if the main purpose of a model is to predict the dynamical behavior of the system, and little significance is attributed to parameter values. This is the case for all models considered by Gutenkunst et al. [5].

Parameter sloppiness becomes much more problematic, however, when models are used explicitly to extract biological information from estimated parameter values. In particular, this affects attempts at reverse engineering gene regulatory networks underlying cellular or developmental processes, where models are used to infer regulatory interactions – and hence regulatory network topology – from quantitative gene expression data.

Identifiability is a mathematical notion. For biological implications the precise values of parameters are not always important as long as they have certain characteristics, like being (roughly) positive, negative or zero. If a posteriori analysis results in a parameter uncertainty range which lies in the characteristic range we call this parameter *determinable*. Note that for those parameters which have to be determined quantitatively, i.e. having no characteristics, determinability refers to a posteriori identifiability.

As a case study, we consider the gap gene system of the vinegar fly *Drosophila melanogaster*. Gap genes constitute the first step in a regulatory cascade that leads to the determination of body segment positions along the major (or anterior-posterior, A-P) body axis during early *Drosophila* development [6]. The biological function of the gap gene system is to interpret long-range protein gradients implemented by the products of the maternal co-ordinate genes (e.g. *bicoid (bcd), hunchback (hb) *and *caudal (cad)*; see [7-9] and references therein). Zygotic gap genes, such as *hb, Krüppel (Kr), knirps (kni) *and *giant (gt)*, are activated or repressed by these maternal gradients, which establishes their expression in broad, overlapping regions of the embryo. These spatial domains of gap gene expression are stabilized and refined by gap-gap cross-repression. In turn, gap genes are involved in regulation of pair-rule and segment-polarity genes, the latter of which establish a segmental pre-pattern of gene expression by the onset of gastrulation.

The gap gene system is one of the best characterized developmental gene networks available today. It has been studied extensively using genetic and molecular approaches (see [7] and references therein). More importantly for our purposes, quantitative expression data are available for all relevant maternal co-ordinate and gap genes [10,11], and those data have been used to infer regulatory interactions between gap genes using different global and local optimization strategies [7,8,12,13]. In this study, we use parameter values from these earlier studies as starting points for local optimization to obtain a large set of parameter estimates. We then apply a practical identifiability analysis to those parameter sets to establish how well these estimates can be determined based on the available experimental data. We discuss the implications our results have for modeling of the gap gene system and for the biological interpretation of estimated parameter values. Finally, we note that the analysis can easily be adapted to other systems, and we strongly recommend its use to systems biology models in which large emphasis is put on the biological interpretation of estimated parameter values.

## Methods

We consider a model given by the system of ODEs of the general form:

(1) $$\{\begin{array}{ll} \frac{\text{d}y}{\text{d}t}=f(t,y,\theta ), & 0<t\leq T,\\ y(t,\theta )=y_{0}(\theta ), & t=0. \end{array}$$

Here the *m*-dimensional vector *θ *contains all unknown parameters, **y **is an *n*-dimensional state vector, and **f **is a given vector function, differentiable with respect to *t*, **y **and *θ*. When components of the initial state vector **y**_0 _are not known, they are considered as unknown parameters. Thus, **y**_0 _may depend on *θ*.

As mentioned above, we assume that (1) is the 'right' model for the problem we are interested in, implying that (1) is a sufficiently accurate mathematical description approximating reality. This means that all relevant knowledge about the modeled processes is incorporated correctly in the vector function **f**. Thus, the only uncertainty in (1) is the vector of unknown parameters *θ*. Furthermore, it means that there exists a 'true' value θ* for the parameters *θ *such that (1) represents reality. Therefore, in principle, all unknown parameters can be determined if sufficient and accurate enough data are available.

Quantities that can be experimentally measured are called *observables*. The theory of identifiability holds in general for observables being a combination of state variables. However, for the sake of simplicity we consider here the particular case when only the components of the state vector are measured. Let us assume that for fitting (1) there are *N *measurements available. Each measurement, which we denote by ${\tilde{y}}_{i}$, is specified by the time *t*_*i *_when the *c*_*i*_-th component of the state vector **y **is measured. The corresponding model value obtained from (1) is denoted by $y_{c_{i}}$(*t*_*i*_, *θ*). The assumptions outlined above imply that the difference $\left|{\tilde{y}}_{i}-y_{c_{i}}(t_{i},\theta^{*})\right|$ is solely due to experimental error. We denote the vector of discrepancies between the theoretical values and the measured values by Y(*θ*). Then the least squares estimate $\hat{\theta }$ of the parameters is the value of *θ *that minimizes the sum of squares [14,15]

(2) $$S(\theta )=\sum_{i=1}^{N}{\left(y_{c_{i}}(t_{i},\theta )-{\tilde{y}}_{i}\right)}^{2}={\text{Y}}^{T}(\theta )\text{Y}(\theta ).$$

We note that (2) is an appropriate measure under certain assumptions only, which we will discuss below. Other measures might be used when these assumptions do not hold.

### Parameter Estimation by the Levenberg-Marquardt Method

There exist a number of different optimization techniques for parameter estimation. The choice of technique usually depends on the type of model equations (deterministic or stochastic), on the number of unknown parameters (moderate or large), as well as on the dependence of model solutions on parameters (linear or nonlinear, continuous or discontinuous). For a survey on optimization methods in biochemical models we refer to [2,16]. In general, model (1) – being nonlinear in *θ *– leads to a least squares problem (2) that has several minima, first because the problem has more than one solution, and second because the fitness function (2) can have several stationary points that do not correspond to the lowest value of the fitness landscape (so-called local minima). *Local search methods*, like Levenberg-Marquardt (LM), easily get trapped in one of the local minima rather than finding the global minimum. To explore the whole search space one needs *global search methods*, like the Evolution Strategy used in [12]. Unfortunately, these methods converge very slowly once near a minimum. In contrast, gradient-based methods are efficient optimizers [17] for nonlinear least-squares problems once a sufficiently good initial guess for the parameter values is available. In this paper we use the solutions from the global search in [12] as initial guesses for local optimization by the LM method [18]. In this way, we reduce the chance of missing the global minimum and the determination of all the minima is precise and fast.

In general, any gradient-based optimization procedure seeks a correction *δθ *for the parameter vector, such that *S*(*θ *+ *δθ*) ≤ *S*(*θ*) holds. The LM method [18] determines the correction as the solution of the equations

(3) (*J*^*T*^(*θ*)*J*(*θ*) + *λI*_*m*_) *δθ *= -*J*^*T*^(*θ*)Y(*θ*),

where *λ *≥ 0 is a control parameter (see below), *I*_*m *_is the identity matrix of size *m *and the Jacobian $J(\theta )=\frac{\partial \text{Y}(\theta )}{\partial (\theta )}$ is the so-called 'sensitivity' matrix of size *N *× *m*. The entry *J*_*i*, *j *_in *J*(*θ*) shows how sensitive the model response is at the *i*-th data point for a change in the *j-th *parameter. The LM method can be seen as the combination of two gradient-based approaches: Gauss-Newton and steepest descent [17]. If *λ *= 0 in (3), it coincides with the Gauss-Newton method. However, when the matrix *J*^*T*^(*θ*)*J*(*θ*) is (almost) singular, to solve (3), *λ *has to be positive and for large *λ *the LM method approaches the steepest descent method. During the optimization *λ *is adapted such that the algorithm strives to exploit the fast convergence of the Gauss-Newton method whenever this is possible [18,19].

In order to solve (3), the singular value decomposition (SVD) [20] of the matrix *J*(*θ*) can be used, i.e.

(4) *J*(*θ*) = *U*(*θ*) Σ (*θ*) *V*^*T*^(*θ*),

where *U*(*θ*) is an orthogonal matrix of size *N *× *m*, such that *U*^*T*^(*θ*)*U*(*θ*) = *I*_*m*_, *V*(*θ*) is an orthogonal matrix of size *m *× *m*, such that *V*^*T*^(*θ*)*V*(*θ*) = *V*(*θ*)*V*^*T*^(*θ*) = *I*_*m*_, and Σ(*θ*) is a diagonal matrix of size *m *× *m *which contains all singular values *σ*_*i *_in non-increasing order. Then the correction *δθ *can be found as

(5) *δθ *= -*V*(*θ*) (Σ^2^(*θ*) + *λI*_*m*_)^-1 ^Σ(*θ*) *U*^*T*^(*θ*) Y(*θ*).

Later, when we study the reliability of the parameters computed, the SVD will play an important role again.

In order to execute an LM optimization step, the vector of discrepancies Y(*θ*), the matrix *J*(*θ*) and its SVD have to be evaluated for each new estimate of *θ*. For this purpose, for Y and the entries of *J *one needs to resolve (1) and the additional system of variational equations (*i *= 1,2,...,*m*)

(6) $$\{\begin{array}{ll} \frac{\partial }{\partial t}\frac{\partial y}{\partial \theta_{i}}=\frac{\partial f}{\partial \theta_{i}}+\frac{\partial f}{\partial y}\frac{\partial y}{\partial \theta_{i}}, & 0<t\leq T,\\ \frac{\partial y(t,\theta )}{\partial \theta_{i}}=\frac{\partial y_{0}(\theta )}{\partial \theta_{i}}, & t=0, \end{array}$$

respectively. We note that the costs for performing the SVD and computing the correction (5) are negligible in comparison with the computational costs for solving (1) and (6).

Thus, a single LM step requires the numerical solution of *m *+ 1 coupled systems, each one consisting of *n *ODEs. Fortunately, these systems are coupled in a special way, namely, for each *i *= 1, 2,...,*m*, system (6) is a system of linear ODEs for $\frac{\partial y}{\partial \theta_{i}}$, coupled only with (1). The system of equations (6) has the same stiffness as (1), so for numerical stability the same step size can be used for the time integration of (1) and (6) (note that ODE stiffness is determined by the eigenvalues of the Jacobian matrix $\frac{\partial f}{\partial y}$ and is not related to parameter stiffness as described above). Therefore, the one-way coupling can be used to solve (1) and (6) efficiently.

Numerical integration of (1) and (6) requires a fast and reliable ODE solver. Search in the parameter space may lead to some values of *θ *such that the systems of ODEs become stiff [21]. It is well known that for stiff ODE systems explicit schemes can give rise to numerical instability or, alternatively, extremely small time steps. Therefore, an implicit scheme is the best choice for time integration for stability reasons. Using an implicit scheme allows us to exploit the specific coupling between (1) and (6) in an efficient way. At each time step *τ *integrating first (1) provides the solution vector **y**. This requires the LU decomposition of the Jacobian matrix $I_{m}-\tau \frac{\partial f}{\partial y}$. Using this LU decomposition the calculation of $\frac{\partial y}{\partial \theta_{i}}$ from (6) reduces to a simple forward substitution and backsubstitution. In our simulations we use a tailor-made code [22] based on the implicit multistep Backward Differentiation Formulas (BDF) [23].

When the unknown parameters have to obey certain constraints – linear or non-linear – some additional work is needed. If the correction *δθ *found by (5) leads to violation of some constraints, then by the introduction of Lagrange multipliers a modified correction can be found, which fits all constraints. For the constrained minimization problem we refer the reader to [22].

For additional modeling and numerical aspects of this method we refer the reader to Additional file 1 (Section 1).

### Statistical Analysis of Parameter Estimates

**Figure 1:**
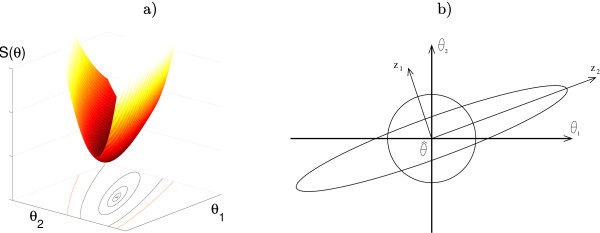
**A graphical representation of the confidence region in the 2-dimensional case**. a) Example of a fitness landscape *S*(*θ*) for a linear model and the contours corresponding to different confidence regions. b) Example of an ellipsoidal confidence region and an accuracy sphere, where principal axes of the ellipsoid, *z*_1 _and *z*_2_, define the new coordinate system which is a rotation of actual parameter space (*θ*_1_; *θ*_2_). Clearly, *z*_1 _is well-determined, while *z*_2 _is not.

Above we used θ* to denote the 'true' parameter vector, for which (1) describes reality with sufficient accuracy, and by $\hat{\theta }$ we denote the parameter vector which minimizes (2). Even having a 'right' model and an estimate $\hat{\theta }$ for the parameter vector which fits the data well, does not mean that the whole modeling problem is resolved successfully. It is important to know how reliable the obtained estimate is. This is the subject of a posteriori identifiability analysis [3,4,24]. One way to look at this is inspecting the fitness landscape *S*(*θ*) in the neighbourhood of $\hat{\theta }$. Roughly speaking, if it is a sharp trough then the true parameter vector θ* and the obtained minimum $\hat{\theta }$ are close. If it is flat in one or more directions, like the surface for a 2-parameter case in Fig. 1(a), then the minimum found can be far apart from the true parameter vector. Near the minimum, where the gradient of *S*(*θ*) vanishes, this surface is approximated by the second derivative or Hessian of *S*(*θ*). If the model is linear in the parameters the Hessian is equal to *J*^*T *^*J*. This linearity assumption and some statistics underlie the following rigorous analysis [14,15,21]. 

We assume that the measurement errors in ${\tilde{y}}_{i}$ are independent of each other and normally distributed and that the error distributions have zero mean and constant standard deviation *σ*. Then, $\hat{\theta }$ is a maximum likelihood estimate [14,15]. By assumption the model with the 'true' solution θ* describes reality, thus

$$\begin{array}{cc} {\tilde{y}}_{i}\approx y_{c_{i}}(t_{i},\theta^{*})+\epsilon_{i}, & i=1,2,...,N, \end{array}$$

where *ϵ*_*i *_are the measurement errors, for which

(7) $$\hat{\theta }-\theta^{*}\sim N_{m}\left(0,\sigma^{2}{\left(J^{T}(\hat{\theta })J(\hat{\theta })\right)}^{-1}\right)$$

holds approximately [14]. Here *N*_*m*_(·,·) denotes the *m*-dimensional multivariate normal distribution. Notice that (7) holds exactly when **y **is linear in *θ*. Next we can define a region around $\hat{\theta }$ in which the 'true' parameter vector θ* lies with a certain probability 1 - *α*. This (1 - *α*)*-confidence region *is determined by the inequality

(8) $${\left(\theta^{*}-\hat{\theta }\right)}^{T}\left(J^{T}(\hat{\theta })J(\hat{\theta })\right)(\theta^{*}-\hat{\theta })\leq \frac{m}{N-m}S(\hat{\theta })F_{\alpha }(m,N-m),$$

where *F*_*α*_(*m*, *N *- *m*) is the upper *α *part of Fisher's distribution with *m *and *N *- *m *degrees of freedom. Geometrically these confidence regions are given by the contours of *S*($\hat{\theta }$) (for linear models), cf. Fig. 1(a).

The ellipsoid defined by (8), is centered at $\hat{\theta }$ and has its principal axes directed along the eigenvectors of *J*^*T*^($\hat{\theta }$)*J*($\hat{\theta }$). Using the SVD (4) for *J*($\hat{\theta }$), we get

$$J^{T}(\hat{\theta })J(\hat{\theta })=V(\hat{\theta })\Sigma^{2}(\hat{\theta })V^{T}(\hat{\theta }),$$

and the eigenvectors of *J*^*T*^($\hat{\theta }$)*J*($\hat{\theta }$) are the columns of the matrix *V*($\hat{\theta }$). So, the ellipsoid has its principal axes directed along the column vectors of the matrix *V*($\hat{\theta }$). Moreover, the radii along these principal axes are inversely proportional to the corresponding singular values *σ*_*i*_, the diagonal elements of Σ($\hat{\theta }$). This all can be seen by using the following transformation (rotation)

(9) $$z=V^{T}(\hat{\theta })(\theta^{*}-\hat{\theta }),$$

yielding

(10) $${\left(\theta^{*}-\hat{\theta }\right)}^{T}\left(V(\hat{\theta })\Sigma^{2}(\hat{\theta })V^{T}(\hat{\theta })\right)(\theta^{*}-\hat{\theta })=z^{T}\Sigma^{2}(\hat{\theta })z=\sum_{i=1}^{m}\sigma_{i}^{2}z_{i}^{2}.$$

On the other hand, since *S*($\hat{\theta }$)/(*N *- *m*) is an unbiased estimator of *σ*^2^, the equation for the ellipsoid can be rewritten as

(11) $$\sum_{i=1}^{m}\sigma_{i}^{2}z_{i}^{2}=r_{\sigma }^{2},$$

where $r_{\sigma }^{2}$ ≈ *mσ*^2^*F*_*α*_(*m, N *- *m*) is proportional to the variance in the measurement errors. This form is more convenient to deal with because **z **can be considered as a set of uncorrelated variables, and once the conclusion has been drawn for the identifiability of **z**, the problem can be transformed back, revealing us the quality of $\hat{\theta }$.

Now, we assume that the model (1) is properly scaled, such that all parameter values are of the same order of magnitudes, and that we are interested only in the first few digits of the parameter values. Let us introduce the sphere given by

$$\sum_{i=1}^{m}z_{i}^{2}=r_{\epsilon }^{2},$$

where *r*_*ϵ *_defines the level of accuracy one desires for the parameter estimates. For instance, if the parameters are of order *O*(1) and one is interested only in the first two digits to the right of the decimal point, then *r*_*ϵ *_= 0.01. In order to be able to determine *z*_*i *_accurately enough, the radius along the ellipsoid's *i*-th principal axis shouldn't exceed the radius of the sphere, which leads us to the following inequality

(12) $$\sigma_{i}\geq \frac{r_{\sigma }}{r_{\epsilon }}.$$

A graphical representation of the ellipsoid and the sphere for the 2-dimensional case is given in Figure 1(b).

If only the first *k *largest singular values satisfy (12), then only the first *k *entries of **z **are estimated with the required accuracy and no sufficient information is available for the remaining components of **z**. Each of the first *k *entries of **z **defines a parameter or a linear combination of parameters which is well-determined. If a principal axis of the ellipsoid makes a significant angle with the axes in parameter space (i.e., there exists more than one significant entry in the eigenvector), this implies correlation between parameters in $\hat{\theta }$.

To summarize, the level of noise in the data in combination with the accuracy requirement for the parameter estimates, defines the threshold for significant singular values in the matrix Σ. The number of singular values exceeding this threshold determines the number of parameter relations that can be derived from the experiment. How these relations relate to the individual parameters is described by the corresponding columns in the matrix *V*. The largest entries in these columns indicate the well-determined parameters. This method is illustrated on the basis of a simple enzymatic reaction in [2].

Finally, (11) indicates that having, for instance, two times more accurate data so that the standard deviation *σ *is halved, will decrease the radii along the ellipsoid's principal axis by a factor of 2. Therefore, in case of very small singular values *σ*_*i *_(i.e. strongly elongated ellipsoids) more accurate data obtained by the experimentalist will not improve the quality of the corresponding parameter estimates by much. In such a case, one certainly needs additional measurements of a different type (e.g. different components, different time points, or in the case of PDEs different spatial points).

Another way of assessing the information from the confidence region is by looking at confidence intervals of the parameter estimates ${\hat{\theta }}_{i}$ (*i *= 1, 2,...,*m*). From (8) one can derive dependent and independent confidence intervals. The *dependent confidence interval *is the intersection of the ellipsoidal region with the *i*-th parameter axis

(13) $$\left\{\theta_{i}:|\theta_{i}-{\hat{\theta }}_{i}|\leq \frac{r_{\sigma }}{\sqrt{{\left(V(\hat{\theta })\Sigma^{2}(\hat{\theta })V^{T}(\hat{\theta })\right)}_{ii}}}\right\},$$

i.e. one assumes that all other parameters are exactly determined. The *independent confidence interval *is the projection of the ellipsoidal region onto the *i-th *parameter axis

(14) $$\left\{\theta_{i}:|\theta_{i}-{\hat{\theta }}_{i}|\leq r_{\sigma }\sqrt{{\left(V(\hat{\theta })\Sigma^{-2}(\hat{\theta })V^{T}(\hat{\theta })\right)}_{ii}}\right\}.$$

Clearly, small independent confidence intervals for ${\hat{\theta }}_{i}$ indicate that it is well-determined. However, in some cases considering only individual confidence intervals can be misleading. For instance, in the presence of strong correlations between parameters, the dependent confidence intervals underestimate the confidence region while the independent confidence intervals overestimate it.

From (7), the covariance matrix of $\hat{\theta }$ is given by

(15) $$\sigma^{2}{\left(J^{T}(\hat{\theta })J(\hat{\theta })\right)}^{-1}=\sigma^{2}V(\hat{\theta })\Sigma^{-2}(\hat{\theta })V^{T}(\hat{\theta }).$$

Then, by denoting $B(\hat{\theta })=V(\hat{\theta })\Sigma^{-2}(\hat{\theta })V^{T}(\hat{\theta })$, the *correlation coefficient *between ${\hat{\theta }}_{i}$ and ${\hat{\theta }}_{j}$ can be computed by

(16) $$\rho_{ij}=\frac{B_{ij}}{\sqrt{B_{ii}B_{jj}}}.$$

We note that by computing individual confidence intervals and correlations between parameters, one is not able to assess the identifiability of linear combinations of parameters. This can be seen only by using the first approach, i.e. by inspection of the *V *and Σ matrix.

### The Biological Test Problem: Gap Gene Circuits

We apply the methodology described above to assess parameter determinability of gene circuit models for the gap gene network in early *Drosophila *development. Here, we provide a brief outline of gap gene circuit models. More detailed information can be found in [7,8,25].

Segment determination occurs during the blastoderm stage of *Drosophila *development, between 1.5 and 3 hours after egg laying [26]. During this stage, the embryo consists of a syncytium; there are no cell membranes between nuclei. These nuclei constitute the basic objects of the model. They are arranged in a row along the A-P axis. Nuclei divide rapidly and synchronously [27]. Periods between mitotic divisions are called cleavage cycles, where cycle *n *occurs between mitoses *n *- 1 and *n*. The models considered here run from early cycle 13 (*t *= 0.0 min) to the onset of gastrulation at the end of cycle 14A (*t *= 71.1 min). Mitosis occurs at the end of cycle 13, between *t *= 16.0 min and *t *= 21.1 min [27].

Gene circuit models describe the change in concentrations of each gap gene product in each nucleus over time by the following system of ODEs

(17) $$\frac{dg_{i}^{a}}{dt}=R_{a}\Phi \left(\sum_{b=1}^{N_{g}}W_{a}^{b}g_{i}^{b}+m_{a}g_{i}^{\text{Bcd}}+h_{a}\right)-\lambda_{a}g_{i}^{a}+D_{a}\left(g_{i+1}^{a}-2g_{i}^{a}+g_{i-1}^{a}\right).$$

*a *and *b *denote regulated genes and regulators respectively. *a *and *b *are integer indices representing *cad, hb, Kr, kni, gt *as well as the terminal gap gene *tailless (tll)*. $g_{i}^{a}$ denotes the concentration of the product of gene *a *in nucleus *i*. The Bcd gradient remains constant over time, and is not regulated by the other genes in the model. $g_{i}^{\text{Bcd}}$ denotes the concentration of Bcd protein in nucleus *i*. *N*_*g *_= 6 is the number of genes in the model (excluding Bcd), and the function

(18) $$\Phi (x)=\frac{1}{2}\left(\frac{x}{\sqrt{x^{2}+1}}+1\right)$$

is a sigmoid regulation-expression function.

During mitosis, protein production is shut down. Nuclei divide instantaneously at the end of mitosis and the distance between them is halved. Gap gene circuits cover the region from 35% to 92% A-P position, which includes 30 (cycle 13) and 58 (14A) nuclei. Therefore, system (17) consists of 180 and 348 ODEs during cycles 13 and 14A, respectively. Initial conditions are prescribed by maternal gradients of Bcd, Cad and Hb, and zero levels for all other gene products. We use no-flux boundary conditions at *i *= 0 and *i *= *i*_max_.

In system (17) there are *m *= 66 unknown parameters. These include the genetic interconnection or regulatory weight matrix *W *of size *N*_*g *_× *N*_*g *_where the matrix elements $W_{a}^{b}$ represent the regulation of gene *a *by gene *b*, while maternal coefficients *m*_*a *_represent the regulatory effect of Bcd on gene *a*. Regulatory parameters represent repression (if < 0), activation (if > 0) or no interaction (if ≈ 0). Other parameters include promoter thresholds *h*_*a*_, promoter strengths *R*_*a*_, diffusion coefficients *D*_*a*_, and decay rates *λ*_*a*_. Estimates for these parameters have been obtained in previous studies by fits to quantitative expression data [11] using global search methods such as parallelized Lam Simulated Annealing [7,8] or the Stochastic Ranking Evolution Strategy (followed by downhill simplex direct search) [12] and using a first-improvement local search method with randomized order of examination [13]. In the latter the initial parameter estimates are obtained by using a splitting strategy: parameters *λ*_*a *_and *D*_*a *_are estimated by assuming that the protein production is constant within certain spatio-temporal domains which reduces (17) to a system of linear equations uncoupled for each gene (the boundaries of production domains are obtained from data); parameters in the nonlinear part of the model are estimated by fitting the production term in (17) with the data given as input, as closely as possible, to the quadrilateral production regions.

**Figure 2:**
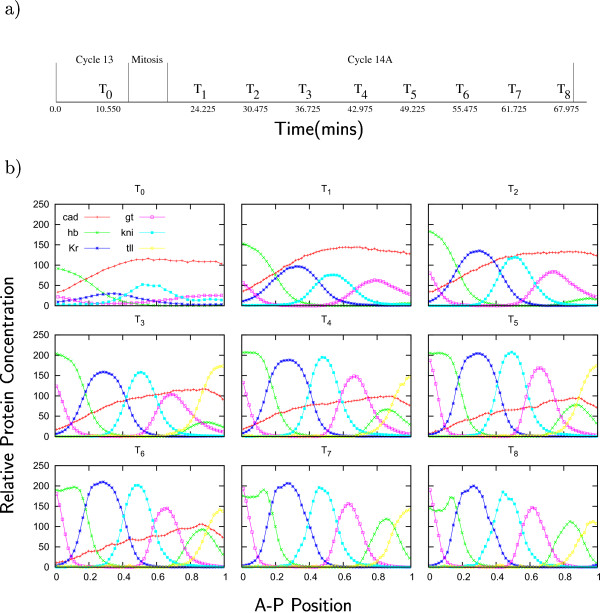
**Data**. a) Time axis and the points when measurements were taken: one in cycle 13 and eight in cycle 14A; the duration of mitosis is also indicated. b) Quantitative gene expression data at different time points. Graphs show relative protein concentration (with a range from 0 to 255 fluorescence units) plotted against position on the A-P axis (the trunk region of the embryo, from 35% to 92% A-P position is scaled to relative co-ordinates [0,1]).

The data set used for model fitting consists of *N *= 2702 measurements of protein concentrations at nuclear resolution (using multi-channel immunofluorescent antibody assays; available online [11]). Measurements were taken at one time point during cycle 13 (*T*_0_), and eight time points *T*_*i *_(1 ≤ *i *≤ 8) during cycle 14A (Figure 2a). Measurements for the concentrations of all gene products represented in the model at all time points are available, except for Cad at *T*_7 _and *T*_8_, and Tll before *T*_3_. The level of measurement error in the data is less than 5%, see [28]. Each data point represents concentration values which have been averaged across 9–62 embryos. Therefore, from the Central Limit Theorem (CLT) we assume that the experimental errors are approximately normally distributed.

The quality of the parameter estimates is measured by the root mean square (*RMS*) of the discrepancy vector

(19) $$RMS(\theta )=\sqrt{\frac{1}{N}\sum_{a=1}^{N_{g}}\sum_{i=1}^{N_{c}}\sum_{j=0}^{N_{t}}\alpha_{j}^{a}{\left(g_{i}^{a}{\left(T_{j},\theta \right)}_{model}-g_{i}^{a}{\left(T_{j}\right)}_{data}\right)}^{2},}$$

where *N*_*t *_= 8 is the number of time classes, *N*_*c *_is the number of nuclei and $\alpha_{j}^{a}$ is equal to zero for Tll at *j *= 0,1, 2 and for Cad at *j *= 7, 8, and is equal to one otherwise. A solution is considered to be 'good' if *RMS *< 12.0 and if there are no visible pattern defects in the model response [7,8,12,13]. It is important to note that the *RMS *only shows the quality of the fit of the model to the data but does not give any information about the quality of the parameter estimates. Our aim is to find the parameter estimates that give a good fit and to apply statistical analysis in order to investigate how reliable these estimates are. 

The search space for parameters is defined by the linear constraints

(20) $$\begin{array}{cccc} 10.0\leq R_{a}\leq 30.0, & 0.0<D_{a}\leq 0.3, & 5.0\leq \frac{ln(2)}{\lambda_{a}}\leq 20.0, & a=1,...,N_{g}, \end{array}$$

and by the nonlinear constraints

(21) $$\begin{array}{cc} \sum_{b=1}^{N_{g}}{\left(W_{a}^{b}g_{max}^{b}\right)}^{2}+{\left(m_{a}g_{max}^{\text{Bcd}}\right)}^{2}+{\left(h_{a}\right)}^{2}\leq 10^{4}, & a=1,...,N_{g}, \end{array}$$

where $g_{max}^{b}$ and $g_{max}^{\text{Bcd}}$ are the maximum values in the data set for proteins *b *and Bcd, respectively. Note that in [7,8,13] threshold parameters *h*_*a *_for genes *Kr, Kni, gt*, and *hb *are fixed to negative values representing a constitutively repressed state for the corresponding genes [7,8]. Fixing some parameters to specific values may severely restrict the search space leaving some solutions out of consideration. Contrary to their approach, we include threshold parameters for these genes in the search by putting the constraints -10.0 ≤ *h*_*a *_≤ 0.0.

In order to make the analysis of parameter estimation easier, we scale all parameters used in (17) in the following way:

$$\begin{array}{cccccc} {\tilde{R}}_{a}=0.1R_{a}, & {\tilde{D}}_{a}=10D_{a}, & {\tilde{\lambda }}_{a}=10\lambda_{a}, & {\tilde{W}}_{a}^{b}=10^{2}W_{a}^{b}, & {\tilde{m}}_{a}=10^{2}m_{a}, & {\tilde{h}}_{a}=h_{a}, \end{array}$$

for all genes *a *and *b*. Note that the choice of the scaling factors for *R*_*a*_, *D*_*a*_, and *λ*_*a *_is based on the search ranges of the corresponding parameters. The choice of the scaling factors for regulatory weights $W_{a}^{b}$ and maternal coefficients *m*_*a *_is based on the fact that the maximum level of protein concentration for all genes in the data set is of order *O*(10^2^). Thus, all scaled parameters are of order *O*(1).

## Results and Discussion

We use 80 different parameter sets, obtained by global search [12], as initial guess for the parameter values and apply the LM method to estimate all 66 unknown parameters of the gap gene circuit model (17), such that the state variables fit the given data (see Figure 2b), subject to (non)linear constraints (20)–(21). Once the parameters are estimated we apply our statistical analysis to assess the quality of the parameter estimates.

### Optimization Results

Least squares estimation of the 66 parameters of the gap gene circuit model (full search case) using the LM method yields a significant decrease of the *RMS *(19) in all simulations (see Table 1). There are only 5 (out of 80) initial parameter sets with *RMS *< 10.0 (best fit: *RMS *= 9.56). After using the LM method there are 71 final parameter sets with *RMS *< 10.0, among which there are 64 with their *RMS *evenly distributed between 8.37 and 9.43. None of these low-scoring parameter sets show any visible patterning defects (see Figure 2.1 in Additional file 1), while most solutions with larger *RMS *do. As it is difficult to make a distinction between these 64 parameter sets based on *RMS *values and expression patterns only, we take all of them into account for our analysis. We note that there is no guarantee that a better solution might have been missed by our parameter estimation procedure. However, since the initial points for the LM search were found by a global search method [12], it is likely that the search space for unknown parameters is explored sufficiently enough.

**Table 1:** RMS distribution for parameter estimates.

	*RMS *< 10.0	10.0 ≤ *RMS *< 12.0	12.0 ≤ *RMS *< 14.0	*RMS *≥ 14.0
*θ*^*in*^	5	36	21	18
${\hat{\theta }}_{full}$	71	3	1	5
${\hat{\theta }}_{fixed}$	63	7	2	8

Entries in the table show the number of parameter estimates with corresponding ranges for *RMS*, where *θ*^*in *^correspond to initial parameter estimate; ${\hat{\theta }}_{full}$ and ${\hat{\theta }}_{fixed}$ correspond to the parameter estimates after using the LM method in the full search case and the case of fixed promoter thresholds, respectively.

**Figure 3:**
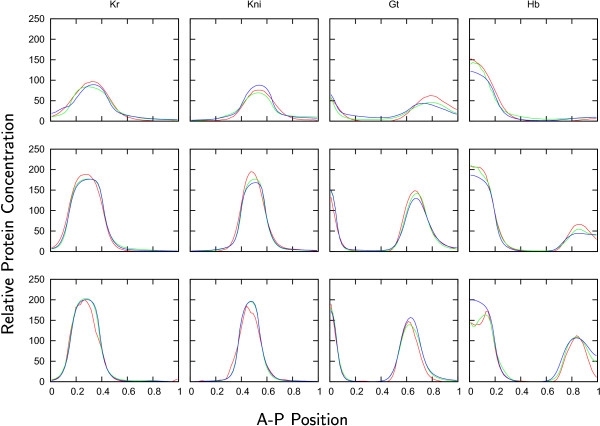
**Model responses vs Data**. Comparison between data (red lines), patterns obtained by a parameter set from [7] (blue lines) and patterns with a parameter set yielded from the LM search (green lines) for the expression of gap genes *Kr, Kni, gt*, and *hb *at early (*t *= 24.225 min, *T*_1_, first row) mid- (*t *= 42.975 min, *T*_4_, second row) and late (*t *= 67.975 min, *T*_8_, last row) cycle 14A. Axes are as in Figure 2.

Parameter estimates found by the LM method significantly improve solution fits found in previous studies (see Figure 3) [7,8,12,13]. However, there are two problems, mentioned in [7,8], that remain unsolved with the new parameter estimates. The first one concerns the artificially high level of gap gene expression during early cycle 13. The model responses are much larger than the data values yielding large positive discrepancies. This is probably due to the lack of protein production delays in the model [7]. The second one concerns the incorrect shift of the posterior Hb domain, which is due to the absence of the terminal gap gene *huckebein (hkb) *from our current models [7,8].

**Figure 4:**
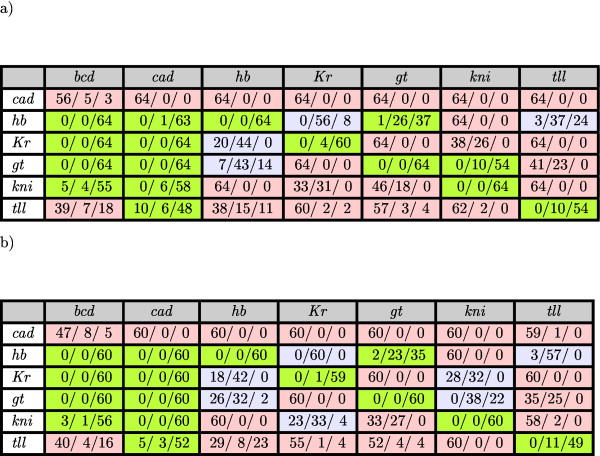
**Regulatory topology of the gap gene network**. Maternal coefficients and regulatory weight matrix for the gap gene system based on parameter sets found by the LM method: a) 64 solutions in the full search case; b) 60 sets in the case of fixed promoter thresholds. Triplets show the number of parameter sets in which a regulatory weight falls into one of the following categories: repression (values ≤ -0.005)/no interaction (values between -0.005 and 0.005)/activation (values ≥ 0.005). Based on the highest value in the triplets, the table is coloured such that the background colours represent activation (green), no interaction (light-blue), or repression (pink).

Many parameters have a broad range of possible values, meaning that they are not uniquely determined (see Figures 2.2-2.3 in Additional file 1). Classification of all parameter estimates for regulatory weights into 'activating', 'repressing' or 'no interaction' categories is shown in Figure 4(a). The resulting network topology is in very good agreement with the results obtained in [7,8,12,13]. Specifically,

(A1) Cad and Bcd activate gap genes *hb*, *Kr*, *kni*, and *gt*;

(A2) gap genes *hb*, *Kr*, *kni*, and *gt *show auto-activation;

(A3) Tll represses gap genes *Kr*, *kni*, and *gt*;

(A4) gap genes with mutually exclusive expression domains strongly repress each other; these correspond to weights $W_{gt}^{Kr}$, $W_{Kr}^{gt}$, $W_{hb}^{kni}$, and $W_{kni}^{hb}$.

Previous results also suggested that pairs of overlapping gap genes (*hb *and *gt*, *gt *and *kni*, *kni *and *Kr*, as well as *Kr *and *hb*) either show no or weak repressive interactions among each other. Note that some of these weights differ slightly from earlier analyses [7,12]. In all of these cases the difference is extremely slight and depends on the threshold chosen to categorize an interaction as 'very weak repression' or 'no interaction' (for example $W_{kni}^{Kr}$ or $W_{Kr}^{kni}$ in Figure 4(a); see also scatter plots in Figure 2.2 in Additional file 1). It is therefore unlikely that such differences are biologically significant. The only activation between overlapping gap genes is predicted for the effect of Gt on *hb*. In addition, we find that Kni activates *gt *in a majority of solutions. In both of these cases, the significance of the interactions does not lie in their weak activating effect (which has no discernible biological function), but rather in the absence of repression [7,8].

### Parameter Determinability

We applied the statistical analysis introduced in the Methods section to the 64 parameter sets obtained by the LM method to assess the quality of our estimates. Ellipsoidal confidence regions corresponding to parameter estimates are given by (10). None of the parameter estimates lies in the ellipsoidal confidence regions of all other parameter sets. Note that this does not necessarily imply that there is no unique 'true' solution for the parameter vector, since the ellipsoidal confidence regions – or at least some of them – may still have a non-empty intersection.

For each parameter set $\hat{\theta }$, the SVD (4) of the Jacobian *J*($\hat{\theta }$) yields the matrices *V*($\hat{\theta }$) and Σ($\hat{\theta }$). In order to find the number of singular values in Σ($\hat{\theta }$) satisfying the accuracy inequality (12), i.e. to determine how many (combinations of) parameters can be determined, we need to quantify *r*_*ϵ *_and *r*_*σ*_. We are interested only in the first decimal digit of the scaled parameters, and therefore we take *r*_*ϵ *_= 0.1. For *α *= 0.05 we obtain *r*_*σ *_≈ 9.4 *RMS*($\hat{\theta }$) (the choice of *α *does not make much difference here due to the large value of *N*).

Investigation of all parameter sets shows that on average, 15 singular values satisfy (12) meaning that at most 15 parameters or linear combinations of them can be determined with one digit accuracy. There is a set of parameters which have significant entries in the first 15 columns of all *V *matrices. It includes regulatory weights $W_{Kr}^{cad}$, $W_{gt}^{cad}$, $W_{kni}^{cad}$, $W_{tll}^{cad}$, $W_{Kr}^{hb}$, promoter thresholds *h*_*Kr*_, *h*_*gt*_, *h*_*tll*_, decay rate *λ*_*cad*_, and promoter strength *R*_*Kr*_. However, inspection of the first 15 columns of the *V *matrices shows that there is not a single parameter which can be determined individually with the chosen accuracy. Thus, each column has a number of significant entries implying that the principal axis of the confidence ellipsoid is at an angle with the corresponding axes in parameter space. This indicates the presence of correlations between parameters.

Dependent and independent confidence intervals for each parameter set can be computed by (13) and (14), respectively. We check if the corresponding confidence intervals for regulatory weights fall entirely into the 'repression', 'no interaction', or 'activation' categories. Results in Figure 4(a) do not change when only dependent confidence intervals are taken into account. However, including independent confidence intervals one can no longer make correct qualitative conclusions about many of the entries in the regulatory weight matrix.

**Figure 5:**
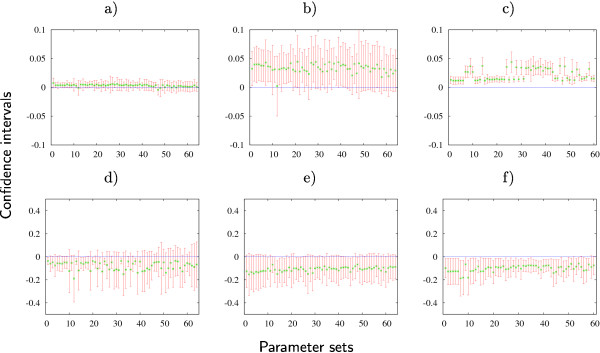
**Confidence intervals for parameter estimates**. Dependent (green lines) and independent (red lines) confidence intervals for regulatory weights $W_{hb}^{Kr}$ (a), $W_{hb}^{cad}$ (b), $W_{kni}^{hb}$ (d), and $W_{gt}^{Kr}$ (e) in the full search case and for regulatory weights $W_{hb}^{cad}$ (c) and $W_{gt}^{Kr}$ (f) in the case of fixed promoter thresholds. Confidence intervals are plotted along the vertical axis for all 64 parameter sets in the full search case and 60 parameter sets in the case of fixed promoter thresholds.

The sizes of the independent confidence intervals give an indication about the determinability of the corresponding regulatory weights. There is a set of eight regulatory weights which have relatively small confidence intervals for all 64 parameter sets (see Figure 2.4 in Additional file 1). It includes $W_{hb}^{Kr}$, $W_{hb}^{tll}$, $W_{Kr}^{hb}$, $W_{Kr}^{Kr}$, $W_{Kr}^{kni}$, $W_{gt}^{hb}$, $W_{gt}^{gt}$, and $W_{gt}^{kni}$. For instance, Figure 5(a) shows the confidence intervals for $W_{hb}^{Kr}$. This regulatory weight is well determined qualitatively, i.e. the independent confidence intervals fall entirely into one category and therefore the type of the regulation can be concluded. The model predicts that Kr does not regulate *hb*. Note that the confidence intervals for these eight parameters in the scaled case are of order *O*(10^-1^) and therefore they are not determinable with the chosen accuracy level *r*_*ϵ *_= 0.1. In fact, they are determinable only if we choose *r*_*ϵ *_= 1.0.

The remaining regulatory weights have larger independent confidence intervals (see Figure 2.4 in Additional file 1) and therefore they are not determined quantitatively. Among them are some regulatory weights for which qualitative conclusions can be deduced from the results. For example, panels (d) and (e) of Figure 5 show the confidence intervals for regulatory weights $W_{kni}^{hb}$ and $W_{gt}^{Kr}$, respectively. Although these two regulatory weights can not be determined quantitatively, there is a qualitative difference between them. The independent confidence intervals for $W_{gt}^{Kr}$ do not extend significantly into the positive part of the plane. Therefore, one can make a qualitative conclusion for this weight: the model predicts that Kr does not activate *gt*. Note that this is a weaker conclusion than predicting repression for this weight from Figure 4(a). In contrast, we cannot draw any qualitative conclusions about $W_{kni}^{hb}$. Thus, our analysis does not confirm the repression of *kni *by Hb inferred from Figure 4(a) (but does not contradict it either). To demonstrate that repression of *kni *by Hb is not strictly necessary to fit the data correctly, we fix this weight to zero while performing parameter estimation. The obtained parameter set has a *RMS *= 9.24 and produces patterns with no visible defects (see Figure 2.7 in Additional file 1).

Based on the confidence intervals, we summarize the qualitative conclusions for the most important regulatory weights in the gap gene system:

(B1) Cad and Bcd do not repress gap genes *hb*, *Kr*, and *gt*; no conclusions can he made for regulation of *kni *by Cad and Bcd;

(B2) gap genes *hb*, *Kr*, *kni*, and *gt *do not show auto-repression;

(B3) Tll does not activate gap gene *gt*; no conclusions can be made for regulation of *Kr *and *kni *by Tll;

(B4) gap genes with mutually exclusive expression domains *gt *and *Kr *do not activate each other; no conclusions can be made for regulatory interactions between *hb *and *kni*.

Interactions between overlapping gap genes are mostly weakly repressive or absent, and are largely consistent with Figure 4(a): confidence intervals for $W_{hb}^{Kr}$, $W_{Kr}^{hb}$, $W_{Kr}^{kni}$, $W_{gt}^{hb}$, and $W_{gt}^{kni}$ indicate no interaction, while confidence intervals for $W_{kni}^{gt}$, and $W_{kni}^{Kr}$ suggest the absence of activation. Finally, confidence intervals for $W_{hb}^{gt}$ indicate the absence of repression.

Obviously, our qualitative conclusions (B1)–(B4) are weaker than the conclusions (Al)–(A4) made from Figure 4(a) by considering only the values of parameter estimates. Note that for all genes, promoter thresholds *h*, promoter strengths *R*, diffusion coefficients *D*, and decay rates *λ *have extremely large independent confidence intervals (see Figure 2.5 in Additional file 1) meaning that all these parameters are not determinable.

**Figure 6:**
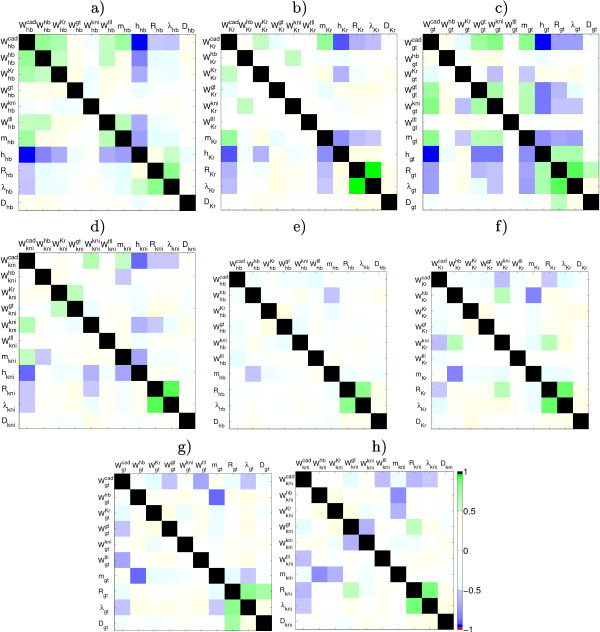
**Correlations between parameters**. Diagonal blocks corresponding to gap genes *hb *(a, e), *Kr *(b, f), *gt *(c, g), and *kni *(d, h) from the mean correlation matrix in the full search case (a, b, c, d) and the mean correlation matrix in the case of fixed promoter thresholds (e, f, g, h).

The large difference between dependent and independent confidence intervals indicates the presence of correlations between parameters. Individual confidence intervals are not informative for understanding the reason of poor determinability of parameters when their estimates are correlated. Using (16), we find the correlation matrix for each parameter set. To detect the most significant correlations between parameters present in all correlation matrices, we calculate an averaged matrix – which we call the mean correlation matrix – whose entries are the mean values of the corresponding correlation coefficients in the individual correlation matrices. The obtained mean correlation matrix has a block diagonal structure such that each block corresponds to a given gene and contains the correlation coefficients between parameters for the same gene (see Figure 2.6 in Additional file 1). Panels (a),(b),(c), and (d) of Figure 6 show the blocks corresponding to gap genes *hb*, *Kr*, *gt*, and *kni*, respectively. Note that the correlations corresponding to the most significant entries in the mean correlation matrix (with absolute values greater than 0.5) are statistically present in all individual correlation matrices because corresponding standard deviations are relatively small (less than 0.2).

### Parameter Estimation with Fixed Promoter Thresholds

The main insight from the mean correlation matrix is that we observe strong correlations of regulatory parameters with promoter thresholds. For instance, regulation of *hb*, *Kr*, *gt*, and *kni *by Bcd and Cad, and auto-regulation are all strongly correlated with their corresponding *h*_*a *_(see panels (a),(b),(c), and (d) of Figure 6). This may explain the poor determinability for these interactions. We checked this hypothesis by fixing promoter thresholds *h*_*a *_for gap genes *hb*, *Kr*, *gt*, and *kni *in (17) to a value of -3.5, similar to the approach used in [7,8,12]. We find that also in this case, least squares estimation using the LM method yields a significant decrease of the *RMS *(see Table 1). There are 63 parameter sets with *RMS *< 10.0 (best fit: *RMS *= 8.66). Among these, there are 60 parameter sets which have no visible patterning defects (see Figure 3.1 in Additional file 1) and these were taken into account in the following analysis. The resulting regulatory network topology (see Figure 4(b)) largely corresponds to that obtained without fixing threshold parameters (full search case) with a few minor exceptions. $W_{kni}^{Kr}$, $W_{Kr}^{kni}$, and $W_{gt}^{kni}$ now all fall into the 'no interaction' category while the full search found mutual repression between *Kr *and *kni*, and activation of *gt *by Kni (compare panels (a) and (b) of Figure 4). As discussed above, these changes represent very small quantitative changes in the parameter values and depend on the (somewhat arbitrary) choice of cut-off between regulatory categories (compare scatter plots in Figures 2.2 and 3.2 in Additional file 1). Therefore, they are unlikely to be biologically significant, while all our main qualitative conclusions (Al)–(A4) on gap gene network topology are fully consistent with our results using fixed threshold parameters.

On the other hand, we observe significant improvement in determinability of some regulatory weights when we compute dependent and independent confidence intervals for each parameter set by (13) and (14), respectively (see Figure 3.4 in Additional file 1). As an example, Figure 5(c) shows the confidence intervals for the regulatory weight $W_{hb}^{cad}$ with fixed promoter thresholds. There is a quantitative improvement in the determinability of this parameter indicated by smaller independent confidence intervals in the case of fixed threshold parameters (compare panels (b) and (c) of Figure 5). But there are also qualitative changes. The model now predicts the activation of *hb *by Cad. Similarly, Figure 5(f) shows the confidence intervals for the regulatory weight $W_{gt}^{Kr}$ with fixed promoter thresholds. Comparison of the panels (e) and (f) of Figure 5 shows that there is no quantitative difference between the two approaches for this weight.

However, we see a qualitative improvement for the case of fixed threshold parameters. The independent confidence intervals in Figure 5(f) lie in the negative part of the plane for almost all parameter estimates and therefore, repression is now predicted for this weight while the plot in Figure 5(e) corresponding to full search case predicts only the absence of activation.

Based on the confidence intervals, we summarize the qualitative conclusions for the essential regulatory weights in the gap gene model in the case of fixed promoter thresholds:

(C1) Cad activates gap genes *hb*, *Kr*, *kni,* and *gt*;

(C2) Bcd does not repress gap genes *hb*, *Kr*, and *gt*; no conclusions can be made for regulation of *kni *by Bcd;

(C3) gap genes *hb*, *Kr*, and *gt *have auto-activation; gap gene *kni *does not have auto-repression;

(C4) Tll does not activate gap gene *gt*; no conclusions can be made for the regulation of *Kr *and *kni *by Tll;

(C5) mutually exclusive gap genes *gt *and *Kr *repress each other; no conclusions can be made for regulations between *hb *and *kni*.

For interaction among overlapping gap genes, the confidence intervals in the case of fixed promoter thresholds are fully consistent with those for the full search case, even though three of these interactions fall into different categories in the analysis based on parameter values only (compare panels (a) and (b) of Figure 4). This shows that confidence intervals can be used to check the significance of ambiguities in predicted interactions based on parameter classification alone. However, although conclusions (C1)–(C5) show qualitative improvement for some regulations in comparison with (B1)–(B4), they are still weaker than those drawn from classifying parameter values only (A1)–(A4).

Similar to the full search case, we compute the mean correlation matrix to detect the significant correlations between parameters (see Figure 3.6 in Additional file 1). The obtained mean correlation matrix also has a block diagonal structure. However, there is a number of significant entries in off-diagonal blocks. Panels (e),(f),(g), and (h) of Figure 6 show the diagonal blocks corresponding to gap genes *hb*, *Kr*, *gt*, and *kni*, respectively. In the absence of dominating correlations between regulatory parameters and thresholds *h*_*a *_we can now identify biologically significant parameter correlations. Here we restrict ourselves to describe some correlations which can be interpreted in biological terms with the emphasis on those for which at least one parameter is 'sloppy':

• Strong negative correlation is present between $W_{kni}^{hb}$ and *m*_*kni*_. That is, strong repression of *kni *by Hb needs to be overcome through increased activation by Bcd. Note that both parameters are poorly determined. In the circuit with $W_{kni}^{hb}$ set to zero, Bcd actually represses *kni *(see Table 2.1 in Additional file 1). This contradicts genetic and molecular evidence indicating that both repression of *kni *by Hb and its activation by Bcd are present in the embryo [29,30].

• There are complex correlations between the (very small, or absent) repressive effects of Hb on *Kr *and *gt*, and the activation of those two genes by Bcd. This confirms earlier results indicating that the balance between activation and repression from maternal genes is crucial for correct gap gene expression [31].

• The importance of the balance between activation and repression is highlighted by the following: repression of *kni *and *gt *by Tll can be compensated through increased activation by Cad, repression of *kni *by Kr can be compensated through increased activation by Bcd, while repression of *kni *by Gt can be overcome by increased *kni *auto-activation in the posterior of the embryo.

• Increased *hb *auto-activation is compensated through decreased activation of *hb *by Bcd indicating that broad maternal activation and auto-regulation are somewhat redundant.

• There is a strong positive correlation present between *m*_*Kr *_and *m*_*gt*_. This correlation is most likely indirect, due to repressive interaction between *gt *and *Kr*. Increased activation of *Kr *by Bcd must be balanced by increased activation of *gt *by Bcd to maintain balance of mutual repression between *Kr *and *gt*.

• There are correlations between activation of *Kr *and *gt *by Bcd and their respective promoter strengths and decay rates. Such correlations are to be expected as stronger expression or increased protein stability can compensate for weaker activation by Bcd.

We note that some of the 'sloppy' parameters, such as $W_{gt}^{Kr}$, $W_{Kr}^{gt}$, $W_{hb}^{kni}$, and $W_{Kr}^{tll}$ are not (strongly) correlated to any of other parameters and their sloppiness remains unclear. The last is completely uncorrelated parameter. Posteriorly Kr is strongly repressed by Gt and somewhat weaker by Hb and Kni. Apparently, due to these interactions repression of Kr by Tll is somewhat redundant in the model.

In summary, the above suggests that complex correlations between regulatory weights as well as correlations between those weights and promoter strength or protein decay rates are an unavoidable property of complex biological networks, as some interactions or changes in expression rate can always compensate for changes in others.

### Parameter Correlations: Data vs Model

Poor determinability of most of the parameters in the gap gene model is due to correlations between parameters. Here we investigate whether these correlations are caused by shortcomings of the data or the model.

At first glance, it seems that insufficient accuracy of the data cannot be the reason for correlations. More accurate data would simply make the ellipsoid confidence region shrink but not rotate. Therefore, it cannot significantly improve the determinability of the parameters (see also [5]). We checked this by assuming that a larger data set was available: Say we had measurements for all gene products, in all nuclei, at 71 uniformly distributed time points (instead of 9). With these choices the total number of measurements would be *N *= 21180. Suppose that we have found that one of our parameter estimates $\hat{\theta }$ minimizes the sum of squares (2). Since the Jacobian depends only on the model responses and not on the values of the data, we can generate a new Jacobian $\tilde{J}(\hat{\theta })$ including all 'ghost' data points. From the SVD of the corresponding $\tilde{J}(\hat{\theta })$ we get the matrices $\tilde{V}(\hat{\theta })$ and $\tilde{\Sigma }(\hat{\theta })$ which define new ellipsoidal regions. The ellipsoids are slightly rotated in comparison with the initial ones but not enough to make the principal axes of the ellipsoid get closer to the parameter axes, i.e. the correlations between parameters are not removed.

Each data point is actually a sample mean, obtained by averaging gene concentrations from individual embryos. Therefore, measurement errors most likely have a normal distribution with zero mean. However, their standard deviations may vary for different data points. Assume that for the *i*-th data point *K*_*i *_measurements from individual embryos are used and assume that the standard deviation of this sample *s*_*i *_is known. Then the normal distribution of the sample mean has a standard deviation which can be estimated by $\sigma_{i}=\frac{s_{i}}{\sqrt{K_{i}}}$. For the dataset we used, both s_*i *_and *K*_*i *_are available from the FlyEx database [11]. Once all *σ*_*i *_are found, we can use a weighted least squares estimation such that *θ *minimizes the sum

$$S(\theta )=\sum_{i=1}^{N}w_{i}^{2}{\left(y_{c_{i}}(t_{i},\theta )-{\tilde{y}}_{i}\right)}^{2}$$

instead of (2). We take the weights *w*_*i *_inversely proportional to *σ*_*i *_such that the weighted least squares yields the maximum likelihood estimate. Also in this case, we find that the obtained parameter estimates have the same type of correlations as those obtained with an ordinary least squares fit (data not shown).

Correlations between parameters can be due to hidden dependencies in the data set. To investigate whether this is the case, we conduct an inverse experiment. We choose one of the parameter sets obtained by the LM search, with an *RMS *= 8.38, and we denote it by θ*. By integrating the model equations with θ* we generate an exact data set at the same data points as the initial data set. To the exact data values we add errors drawn from the normal distribution with zero mean and standard deviation equal to 8.5. From the exact and the perturbed data set, we compute *RMS*(θ*) = 8.17. The perturbed dataset is used for the parameter estimation by means of the LM search. By constructing this inverse problem, we make sure that the assumption about the independence of the measurement errors is correct. With 40 different initial values of *θ *from [12] we obtain 34 parameter estimates having *RMS *between 7.95 and 8.25. Inspection of the corresponding *V *matrices shows that parameters are not determinable due to the correlations, similar to the original problem.

We conclude that the observed correlations between parameters are a property of the model, not of the data. Since an explicit form of the dependence of the state vector on the parameters is not known, the use of reparametrization techniques is not feasible. Note that the majority of parameters in (17) appear in the argument of the sigmoid regulation-expression function Φ. If the model (17) is used to obtain only qualitative information, such as the signs of regulatory weights, then the particular mathematical form of this function is of no importance [25]. However, it has to be studied if the choice of the sigmoid function affects the determinability of parameters.

## Conclusion

In this paper we have applied the Levenberg-Marquardt (LM) optimization method to obtain a set of parameter estimates for gap gene circuit models. We then used statistical analysis to study the quality of these estimates, i.e. how well the parameters are determined with the available experimental data. Our analysis shows that none of the model parameters can be determined individually with reasonable accuracy due to correlations between parameters. Therefore, current gene circuit models cannot be used as a tool to infer quantitative regulatory weights for the gap gene network.

With this caveat in mind, however, it is still possible to draw qualitative conclusions on the regulatory topology of the gap gene network. These conclusions are weaker than, but entirely consistent with those made by only considering the values of parameter estimates [7,8,12,13]. Therefore, they are also fully consistent with genetic and molecular evidence on gap gene regulation (see [7], and references therein). Our analysis allows us to determine exactly which interactions predicted by gene circuit analysis remain ambiguous. If considered in isolation, this ambiguity poses a serious problem for inferring regulatory interactions from expression data as it leaves important aspects of gap gene regulation unresolved. We show that more and better data will not necessarily improve parameter estimates. On the other hand, our results using fixed threshold parameters indicate that at least some of these ambiguous aspects can be resolved by reducing parameter correlations through fixing some parameters in the optimization. Others may disappear if more realistic models are used: for instance, models incorporating protein production delays, or reduced models incorporating *cad *and *tll *as time-variable external inputs as these genes are not regulated by gap genes themselves. Further research into parameter correlations within complex network models will be required to explore what kind of improved models or optimization constraints lead to better parameter determinability.

Still it remains doubtful whether an approach can be found which leads to complete parameter determinability. The study by Gutenkunst et al. [5] indicates that parameter sloppiness is a very common phenomenon among models used in systems biology. Our results corroborate this as it is difficult to see how, for example, correlations between regulatory weights could be eliminated from a network model. The situation is not hopeless, however, as genetic evidence can help us clarify these remaining ambiguous interactions. Such evidence is itself ambiguous in many cases, as it is often difficult to interpret mutant phenotypes. But it is also complementary to and completely independent of the evidence gained by reverse engineering approaches such as the one used here [7]. This means that its ambiguities are often complementary to the ones described in this study. For instance, while cross-repressive feedback between *hb *and *kni *is not supported (but also not contradicted) by our current models, it is very strongly supported by genetic evidence [30]. Based on this, we conclude that systems biology approaches are most successful if they combine experimental and theoretical insights in a consistent and powerful manner.

Other biological interpretations of parameter sloppiness are possible. Our results on the interactions between *hb *and *kni *indicate that although present in the *Drosophila* embryo, they are not strictly necessary to maintain correct gap gene expression, and may be at least partially redundant with or replaceable by other regulatory interactions in the system. It is interesting to think about this from an evolutionary point of view, as such redundancy or replaceability allows the network to be re-wired while maintaining correct gap gene expression.

## Authors' contributions

MA performed the optimization and the statistical analysis; JB proposed the research and supervised the work; JJ contributed to the biological interpretation of the results. All authors contributed to the manuscript.

## Supplementary Material

Additional file 1 **Supplementary Material**. This file (supplment.pdf) contains the material which is not given in the paper due to the space limitations. This file consists of three parts. Section 1 outlines some technical aspects of the methodology described in the paper. Section 2 presents the results obtained in the full search case. It includes plots for model responses compared to the data, scatter plots of parameters, plots of the confidence intervals for parameter estimates, discussion about the correlations between parameters, and results of parameter estimation with fixed weight for regulation of *kni *by Hb. Section 3 presents the results in the case of fixed promoter thresholds, including plots for model responses compared to the data, scatter plots of parameters, plots of the confidence intervals for parameter estimates, and discussion about the correlations between parameters. Click here for file
