# Supplementary material for: Parameter estimation and determinability analysis applied to Drosophila gap gene circuits
Source: BMC Syst Biol. 2008 Sep 25;2:83. doi: 10.1186/1752-0509-2-83 (PMC2586632; doi:10.1186/1752-0509-2-83)
Supplement: Additional file 1 — Supplementary Material. This file (supplment.pdf) contains the material which is not given in the paper due to the space limitations. This file consists of three parts. Section 1 outlines some technical aspects of the methodology described in the paper. Section 2 presents the results obtained in the full search case. It includes plots for model responses compared to the data, scatter plots of parameters, plots of the confidence intervals for parameter estimates, discussion about the correlations between parameters, and results of parameter estimation with fixed weight for regulation of kni by Hb. Section 3 presents the results in the case of fixed promoter thresholds, including plots for model responses compared to the data, scatter plots of parameters, plots of the confidence intervals for parameter estimates, and discussion about the correlations between parameters. [file 1752-0509-2-83-S1.pdf]

Supplementary material for the paper

Parameter estimation and determinability analysis applied to *Drosophila* gap gene circuits

M. Ashyraliyev*, J. Jaeger and J. G. Blom

1 Technical aspects

Here, we outline some general remarks one should be aware of to apply the methodology in practice.

1. If the model is given by a system of Partial Differential Equations (PDEs), then by applying a spatial discretization, it can be reduced to the system of Ordinary Differential Equations (ODEs) (1)[1)]. However, in such a case one has to be careful with the choice of the grid size of the spatial discretization. On the one hand, the grid should be fine enough, so that the numerical errors introduced by spatial discretization are negligible in comparison with the level of noise in the data. On the other hand, requiring an extremely fine grid would increase the size of the system (1). The latter may be crucial in terms of computational complexity.

2. When the model includes algebraic equations, the systems of ODEs (1) and (6) change to Differential Algebraic Equations (DAEs). Since we use an implicit solver for the time integration, the method we have described is readily applicable for that type of models.

3. Given $\mathbf{f}$ and $\mathbf{y}_0$ in (1), the partial derivatives $\frac{\partial \mathbf{f}}{\partial \mathbf{y}}$, $\frac{\partial \mathbf{f}}{\partial \theta_i}$, $\frac{\partial \mathbf{y}_0}{\partial \theta_i}$ $(i = 1, \dots, m)$ in (6) can be, in principle, found analytically. However, for large scale problems when $\mathbf{f}$ has a complicated nonlinear form, this can be a tedious work to do. In such cases, these derivative functions can be generated automatically by using a symbolic mathematics package, like *Maple* [1] or *Mathematica* (Wolfram Research, Inc).

4. Numerically solving (6) has limitations for large scale problems due to computational costs. Another approach to approximate the matrix $J(\theta)$ could be by means of divided differences. The j-th column of $J(\theta)$ is then given by

$$\frac{\partial \mathbf{Y}(\theta)}{\partial \theta_j} \approx \frac{\mathrm{Y}(\tilde{\theta}^j) - \mathrm{Y}(\theta)}{\delta \tilde{\theta}_j},$$

* *E-mail address:* M.Ashyraliyev@cwi.nl

1) Throughout this file we directly refer to equations used in the main paper

where the vector $\tilde{\theta}^j$ is obtained by a small perturbation $\delta\tilde{\theta}_j$ in the j-th entry of θ. In this case, for one Levenberg-Marquardt (LM) step system (1) has to be numerically integrated $m+1$ times. With regard to the computational costs, when $\mathbf{f}$ is non-linear, it is more expensive than the approach where the linear systems of variational equations are solved. Another drawback of the divided difference method is that the numerical approximations introduce additional errors.

5. For large scale problems computation on a single processor can become unfeasible and one needs to use a parallel machine. Parallelization of the computational work when (1) and (6) are solved numerically is only possible at the level of the time step of the integrator. Therefore, it will be inefficient due to heavy communication. The advantage of the divided difference approach is that in this case (1) is solved for $m + 1$ different values of θ independently of each other. Therefore, parallelization of the computational work by divided difference method is trivial and can be very efficient.

6. Variational equations (6) coupled with (1) result in a system consisting of $m+1$ subsystems of the size n. The Jacobian of the coupled system has the following form:

$$\begin{pmatrix} \frac{\partial \mathbf{f}}{\partial \mathbf{y}} & 0 & \dots & 0 \\ \frac{\partial^2 \mathbf{f}}{\partial \theta_1 \partial \mathbf{y}} + \frac{\partial^2 \mathbf{f}}{\partial \mathbf{y}^2}\frac{\partial \mathbf{y}}{\partial \theta_1} & \frac{\partial \mathbf{f}}{\partial \mathbf{y}} & 0.. & 0 \\ \vdots & 0 & \ddots & 0 \\ \frac{\partial^2 \mathbf{f}}{\partial \theta_m \partial \mathbf{y}} + \frac{\partial^2 \mathbf{f}}{\partial \mathbf{y}^2}\frac{\partial \mathbf{y}}{\partial \theta_m} & 0 & ..0 & \frac{\partial \mathbf{f}}{\partial \mathbf{y}} \end{pmatrix}$$

The structure of the Jacobian reveals the one-way coupling of the system. Using an implicit scheme we can exploit this specific coupling between (1) and (6) in an efficient way. At each time step τ integrating first (1) provides the solution vector $\mathbf{y}$. This requires the LU decomposition of $I_m - \tau\frac{\partial \mathbf{f}}{\partial \mathbf{y}}$. Using this LU decomposition the calculation of $\frac{\partial \mathbf{y}}{\partial \theta_i}$ from (6) reduces to a simple forward substitution and backsubstitution.

2 Results of parameter estimation in the full search case

The mathematical model of pattern formation of gap genes in *Drosophila melanogaster*, given by (17), includes 66 unknown parameters. These include the regulatory weight matrix W of size 6×6 with the entries W_a^b representing the regulation of gene a by gene b, six maternal coefficients m_a representing the regulatory effect of Bcd on gene a, six promoter thresholds h_a, six promoter strengths R_a, six diffusion coefficients D_a, and six decay rates λ_a.

In the full search case, all 66 parameters are estimated such that the model fits the given data set. The search space for parameters is defined by the linear and nonlinear constraints given by (19) and (20), respectively. Additionally, the linear constraints $-10.0 \leq h_a \leq 0.0$ are used for promoter thresholds of gape genes hb, Kr, gt, and kni.

Starting with 80 different initial values for the parameter vector from [2], the least squares estimation using the LM method yields 64 parameter sets having no significant patterning defects for the expression of gap genes. In Figure 2.1 the model responses for all 64 parameter sets (green lines) are compared to the data (red lines) at nine time points T_i $(0 \leq i \leq 8)$

when measurements were taken. The model responses for the obtained parameter sets have the same quality and therefore, in our analyses, we have taken into account all of them.

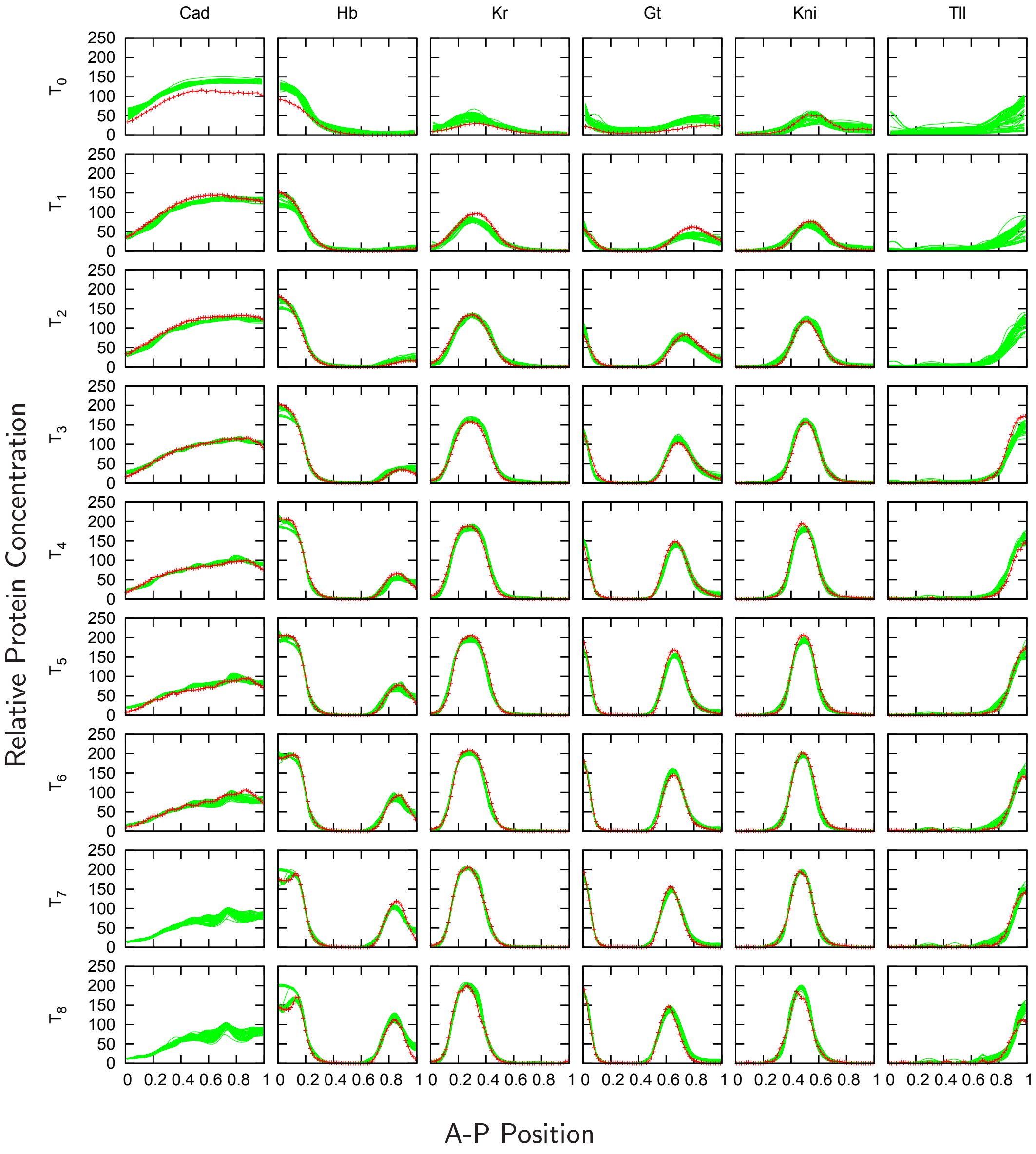

Figure 2.1: Comparison between data (red lines) and the patterns obtained by parameter sets yielded from the LM search (green lines). Graphs show relative protein concentration (with a range from 0 to 255 fluorescence units) plotted against position on the A–P axis (the region of interest from 35% to 92% A–P position is scaled to $[0, 1]$)

Scatter plots in Figures 2.2-2.3 show the range of the estimated parameter values. For

each individual parameter indicated on the horizontal axis, its estimated values (red circles) are plotted along the vertical axis. Most of the parameters have a broad range of possible values, meaning that they are not uniquely found. The only exceptions are some entries in the regulatory weight matrix, such as W^{cad}_{kni}, W^{hb}_{Kr}, W^{hb}_{gt}, W^{Kr}_{hb}, W^{gt}_{gt}, W^{kni}_{gt}, W^{tll}_{cad}, and W^{tll}_{hb}. Note that for some of the regulatory weights all estimated values lie either on positive or negative part of the plane irrespective of width of the range of possible values. So, based only on estimated values, one can make qualitative conclusions (A1)-(A4) (see paper) about the type of the regulation for corresponding weights, i.e activation or repression. Our results are in good agreement with the results obtained in [2, 3, 4].

2.1 Confidence intervals of parameter estimates in the full search case

We compute dependent and independent confidence intervals for each obtained parameter set by (13) and (14), respectively. The sizes of the confidence intervals give the indication about the determinability of corresponding parameters. Figure 2.4 shows the confidence intervals for all regulatory weights and maternal coefficients in the gap gene model. Dependent (green lines) and independent (red lines) confidence intervals are plotted along the vertical axis for all 64 parameter sets. Small dependent confidence intervals in comparison with corresponding independent confidence intervals indicate the presence of correlations between parameters. In such case considering only dependent confidence intervals is not sufficient and therefore we base our conclusions only on independent confidence intervals.

Notice the difference in the order of magnitude of the independent confidence intervals for different parameters. It means that parameters qualitatively differ from each other in terms of determinability. There is a set of eight regulatory weights which have relatively small confidence intervals for all 64 parameter sets. It includes the regulatory weights W^{Kr}_{hb}, W^{tll}_{hb}, W^{hb}_{Kr}, W^{Kr}_{Kr}, W^{kni}_{Kr}, W^{hb}_{gt}, W^{gt}_{gt}, and W^{kni}_{gt}. The remaining regulatory weights have larger confidence intervals. Despite of it, for some regulatory weights, it is possible to make qualitative conclusions about the type of the corresponding regulation. For instance, independent confidence intervals for regulatory weight W^{Kr}_{gt} do not extend significantly to the positive part of the plane. Therefore, one can make the conclusion that Kr does not activate *gt*, i.e Kr represses *gt* or does not regulate it.

Based on the confidence intervals, we get the qualitative conclusions (B1)-(B4) for the essential regulatory weights in the gap gene system. However, the obtained qualitative conclusions are weaker than the conclusions made by considering only the values of parameter estimates.

Finally, we note that promoter thresholds h, promoter strengths R, diffusion coefficients D, and decay rates λ for all genes have extremely large independent confidence intervals (see Figure 2.5). So, all these parameters are nondeterminable.

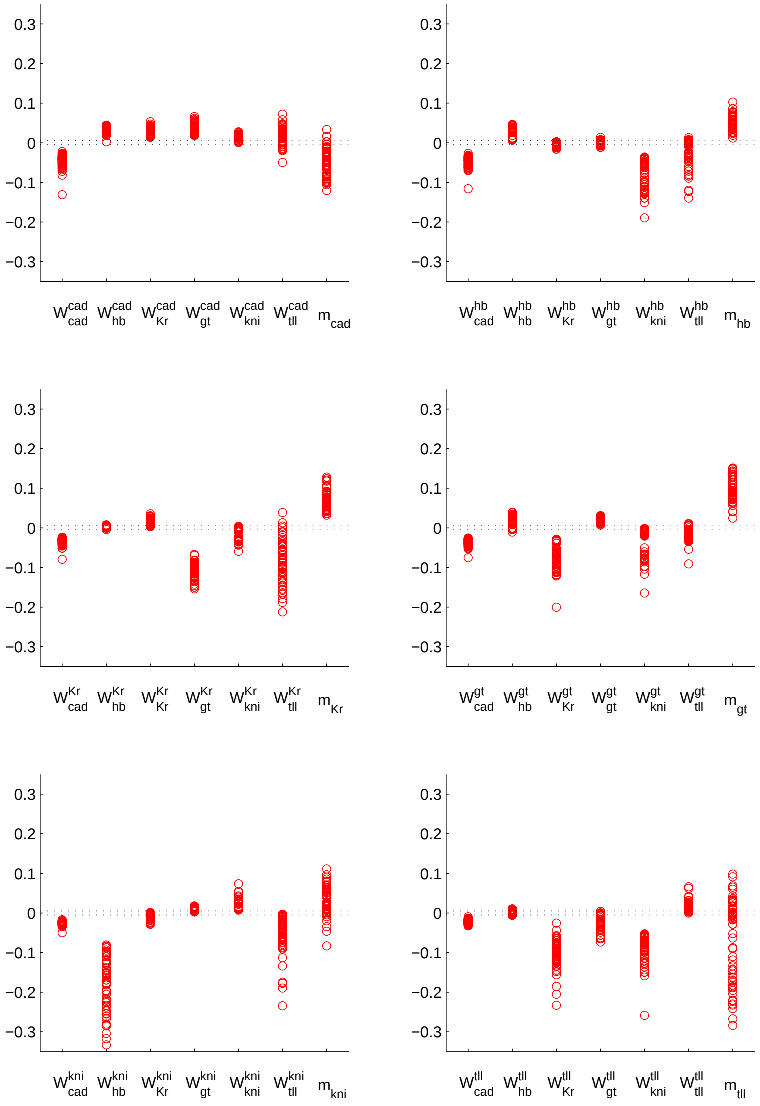

Figure 2.2: Scatter plots of regulatory weights W and maternal coefficients m (full search case).

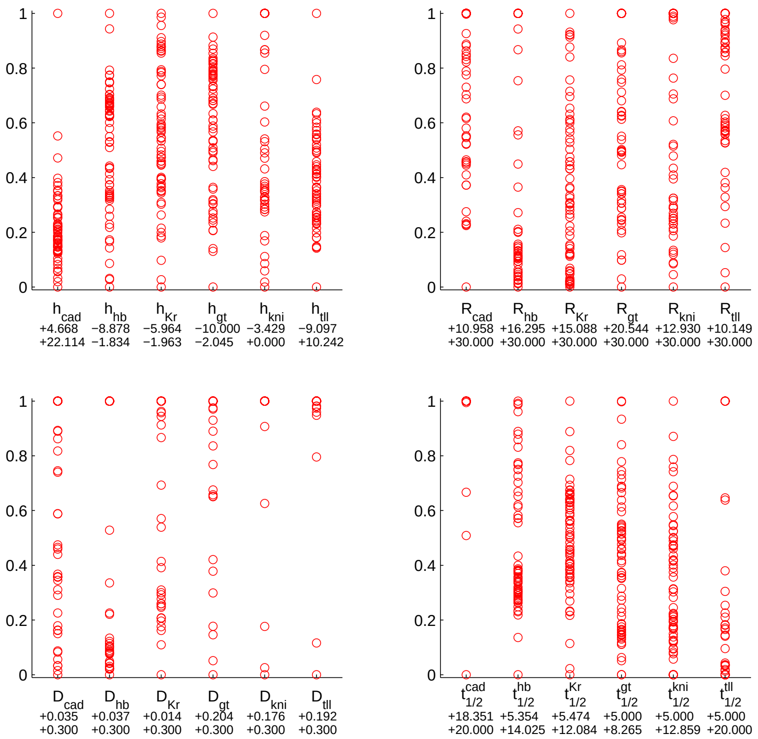

Figure 2.3: Scatter plots of parameters h, R, D and $t_{1/2} = ln(2)/\lambda$ (full search case).

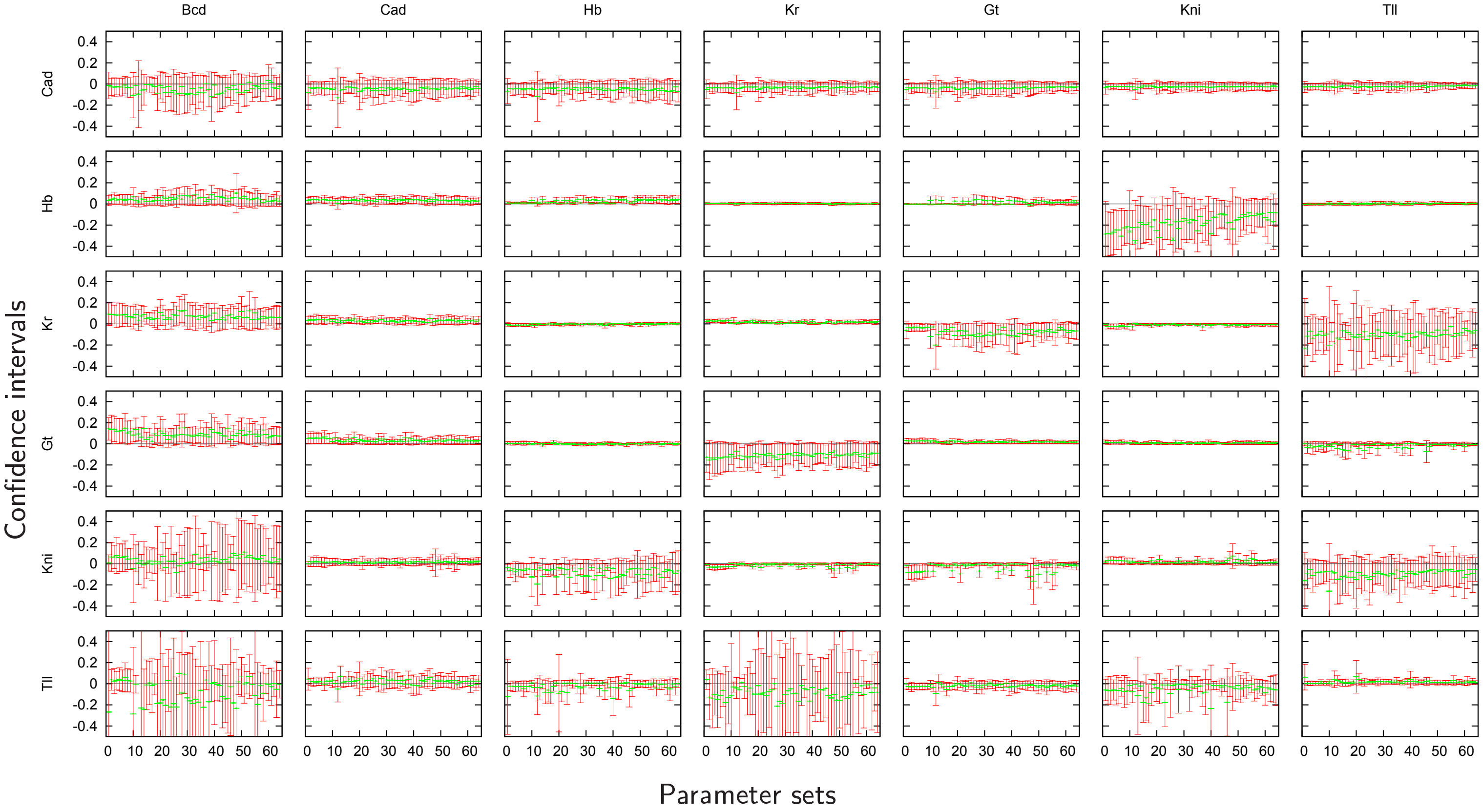

Figure 2.4: Confidence intervals for regulatory weights and maternal coefficients (full search case).

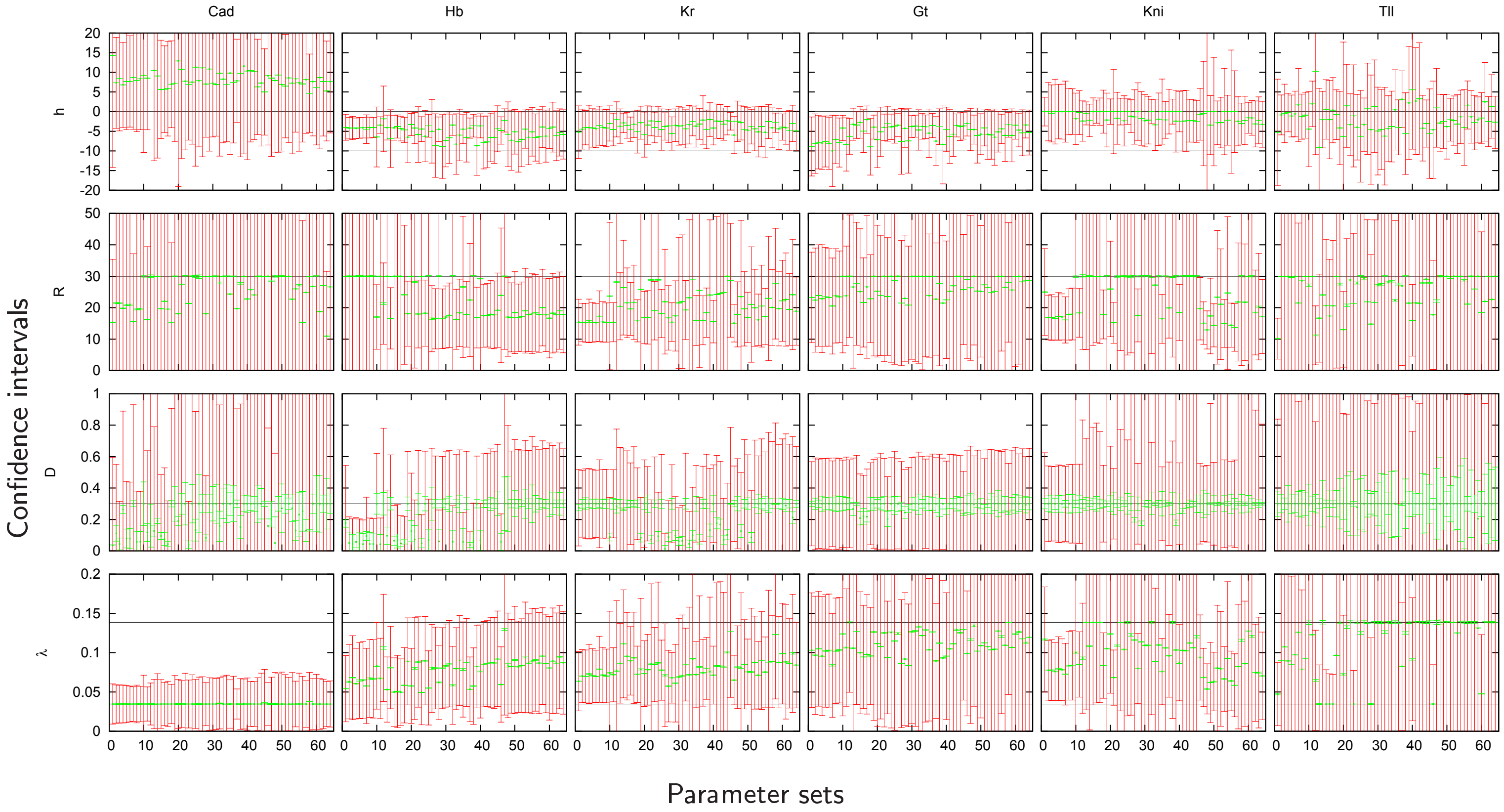

Figure 2.5: Confidence intervals for parameters h, R, D and λ (full search case).

2.2 Correlations between parameters in the full search case

By using formula (16), we find the correlation matrix for each parameter set. To detect the most significant correlations between parameters present in all correlation matrices, we calculate the averaged matrix, which we call the mean correlation matrix, whose entries are mean values of corresponding correlation coefficients in the individual correlation matrices. Figure 2.6 shows the mean correlation matrix. The obtained mean correlation matrix has a block diagonal structure such that each block corresponds to a given gene and contains the correlation coefficients between parameters which enter in the model equations for the same gene.

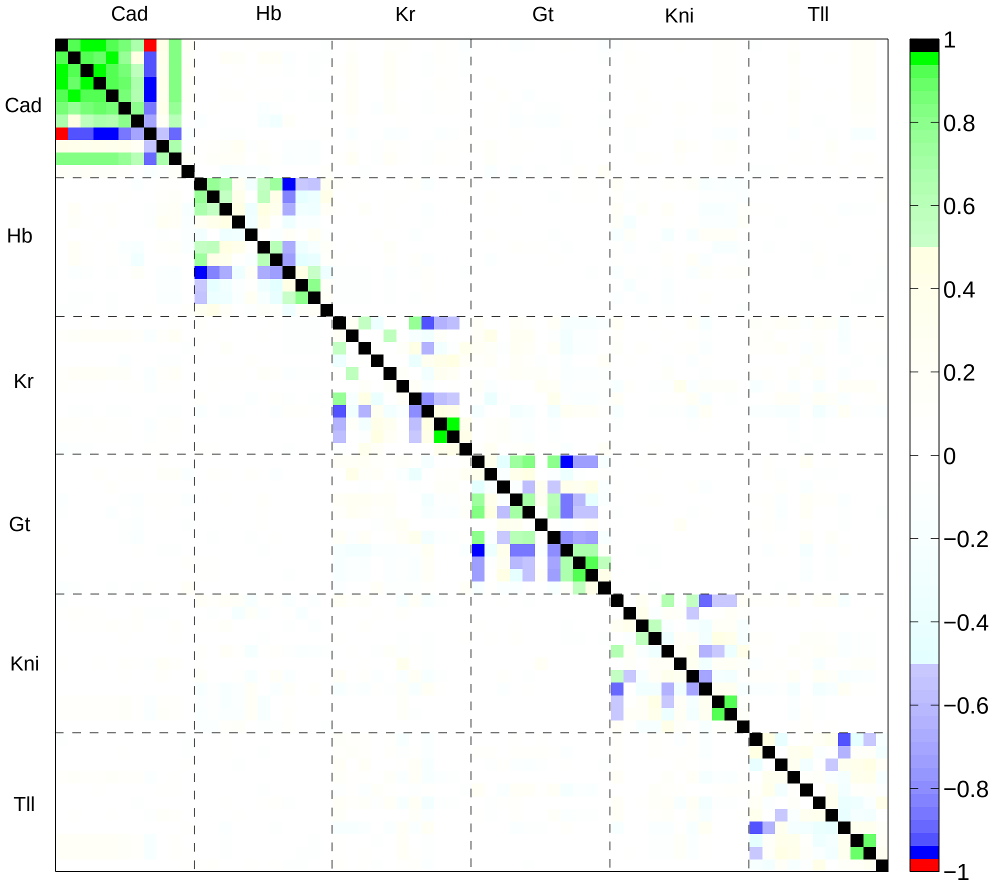

Figure 2.6: Mean correlation matrix (full search case).

A closer look at the most significant correlations in the blocks corresponding to gap genes *hb*, *Kr*, *gt*, and *kni* (see panels (a,b,c,d) of Figure 6 in the paper) reveals that for these genes the regulation by Cad, the regulation by Bcd, and auto-regulation are all strongly correlated with their corresponding promoter threshold h, which may explain the poor determinability for these regulations.

Note that the correlations corresponding to the most significant entries in the mean correlation matrix (with absolute values greater than 0.5) are statistically present in all individual correlation matrices because corresponding standard deviations are relatively small (less than 0.2).

Parameters entering in the model equations for *cad* are all correlated with each other except D_{cad} which seems to be completely uncorrelated. Similar to it, parameters from the model equations for *tll* are also correlated with each other though these correlations somewhat weaker in comparison with correlations between parameters for *cad*. Correlations and therefore nondeterminability of the parameters in the model equations for *cad* and *tll* may stem from the fact that they are not regulated by gap genes [6].

Finally, we note that for all genes promoter strengths R_a are strongly correlated with the corresponding decay rates λ_a meaning that the change in the production is compensated by the change in decay in the model. Strong correlations between them are likely to be the reason for the nondeterminability of these parameters.

2.3 Results of parameter estimation with $W^{hb}_{kni} = 0$

Repression of *kni* by Hb is well-known. In order to show that this regulation is not necessarily required for the gap gene model to fit the given dataset, we perform parameter estimation by using LM method with $W^{hb}_{kni} = 0$, fixed during the search. The obtained parameter set has $RMS = 9.24$ and produces correct patterns. In Figure 2.7 the model response for obtained parameter set (green lines) is compared to the data (red lines). Table 2.1 gives estimated values for parameters.

	cad	*hb*	*Kr*	*gt*	*kni*	*tll*
			**Regulatory matrix**			
cad	-0.030960	-0.042535	-0.033565	-0.031222	-0.019038	-0.020498
hb	0.042596	0.017928	0.007505	-0.001396	-0.164417	-0.000023
Kr	0.050071	0.006310	0.026545	-0.037528	-0.008158	-0.095337
gt	0.045165	0.013300	-0.082837	0.022023	0.009215	-0.044591
kni	0.048618	0.000000	-0.025891	-0.103259	0.056123	-0.097551
tll	0.026991	-0.044042	-0.071046	-0.022854	-0.095755	0.009204
			**Other parameters**			
m	0.004999	0.017453	0.029278	0.032057	-0.013666	0.014765
h	6.494384	-4.945290	-5.836957	-6.032681	-4.167530	-1.538291
R	14.514200	18.666910	17.012730	27.507000	14.958820	21.700990
D	0.300000	0.061481	0.300000	0.300000	0.300000	0.300000
$t_{1/2}$	20.000000	12.501420	9.243277	5.913411	10.398300	13.591250

Table 2.1: Parameter values obtained by LM method for gap gene model with $W^{hb}_{kni} = 0$.

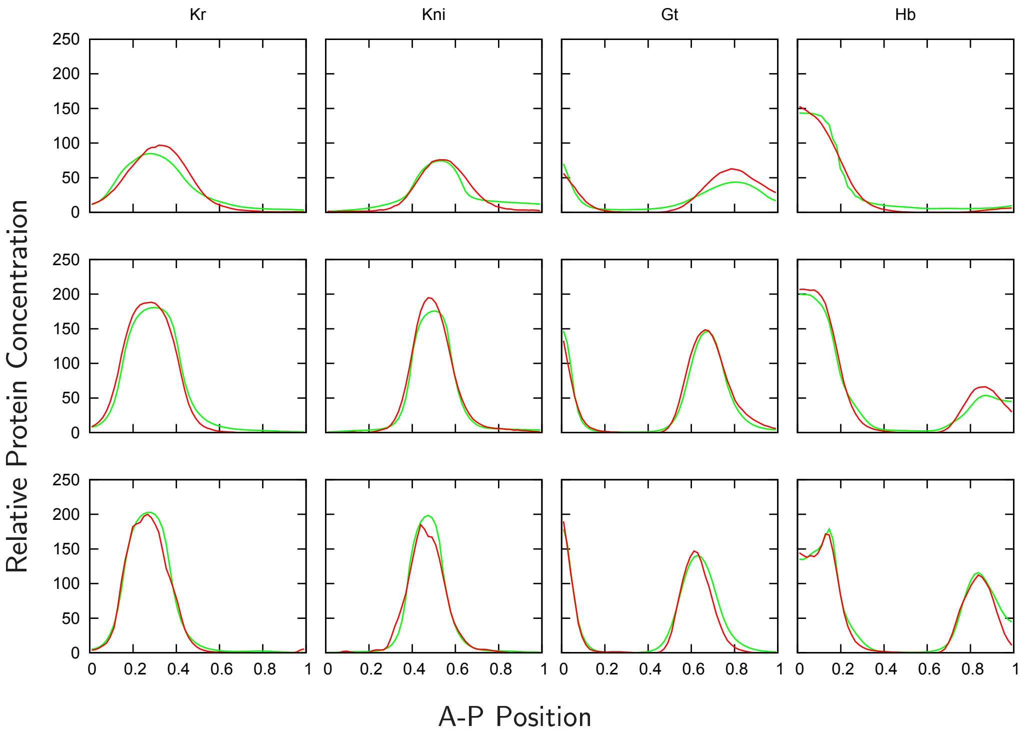

Figure 2.7: Comparison between data (red lines) and patterns obtained by parameter set with $W^{hb}_{kni} = 0$ (green lines) for the expression of gap genes Kr, Kni, gt, and hb at T_1 (first row), T_4 (second row), and T_8 (last row).

3 Results of parameter estimation in the case of fixed promoter thresholds

In the gap gene model (17), we fix four promoter thresholds h for gap genes hb, Kr, gt, and kni to the value -3.5, similar to the approach used in [2, 3, 4]. Then the remaining 62 parameters are estimated by using the LM method.

Similar to the full search case, parameters are estimated such that the model fits the given data set and the search space for parameters is defined by the linear and nonlinear constraints given by (19) and (20), respectively. Starting with the same 80 initial values for the parameter vector as in the full search case, the least squares estimation using the LM method yields 60 parameter sets having no significant patterning defects for the expression of gap genes. In Figure 3.1 the model responses for all 60 parameter sets (green lines) are compared to the data (red lines) at all time points when measurements were taken. Graphs show gene concentrations (along y-axis) plotted against the position on the A–P axis.

The model responses for the obtained parameter sets have the same quality and therefore, in our analyses, we take into account all of them. Note that qualitatively the patterns obtained in the fixed case (Figure 3.1) are comparable to corresponding patterns produced with parameter estimates in the full search case (see Figure 2.1).

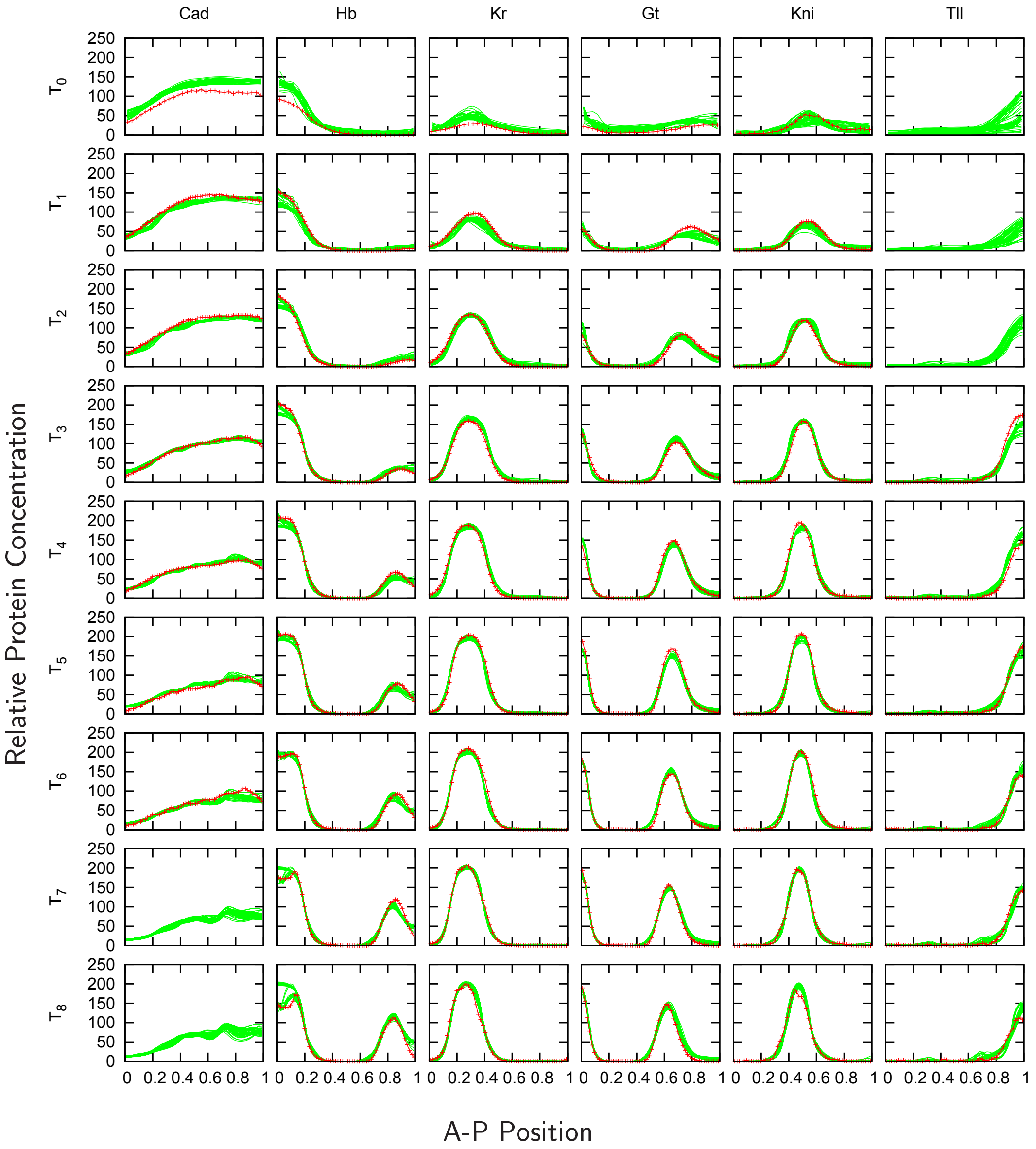

Figure 3.1: Comparison between data (red lines) and the patterns obtained by parameter sets yielded from the LM search (green lines). Axes are as in Figure 2.1.

Scatter plots in Figures 3.2-3.3 show the range of the estimated parameters in the fixed case. For each individual parameter indicated on the horizontal axis, its estimated values (red circles) are plotted along the vertical axis.

In addition to eight regulatory weights which have small range of the values in the full search case, there are nine regulatory weights having the same property in the fixed case,

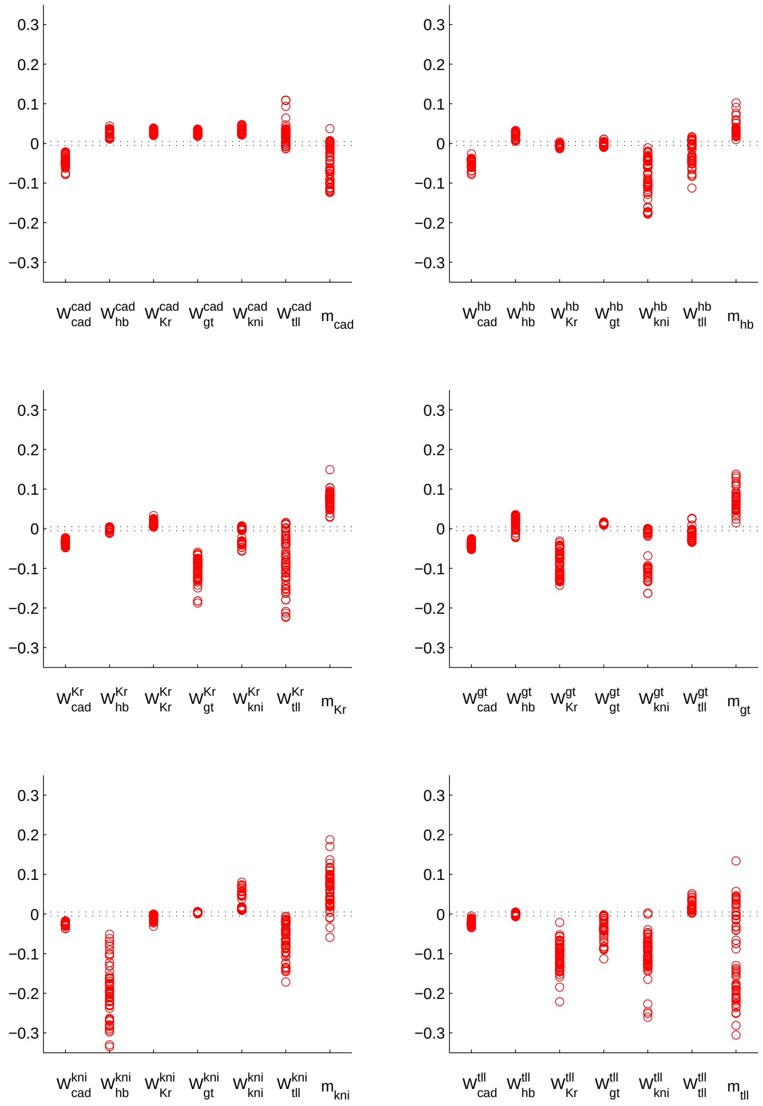

Figure 3.2: Scatter plots of regulatory weights W and maternal coefficients m (fixed case).

such as W^{cad}_{hb}, W^{cad}_{Kr}, W^{cad}_{gt}, W^{hb}_{hb}, W^{Kr}_{cad}, W^{Kr}_{Kr}, W^{gt}_{cad}, W^{kni}_{cad}, and W^{kni}_{Kr}. The remaining parameters have a broad range of values.

3.1 Confidence intervals of parameter estimates in the case of fixed promoter thresholds

We compute dependent and independent confidence intervals for each obtained parameter set by (13) and (14), respectively. Figure 3.4 shows the confidence intervals for regula-

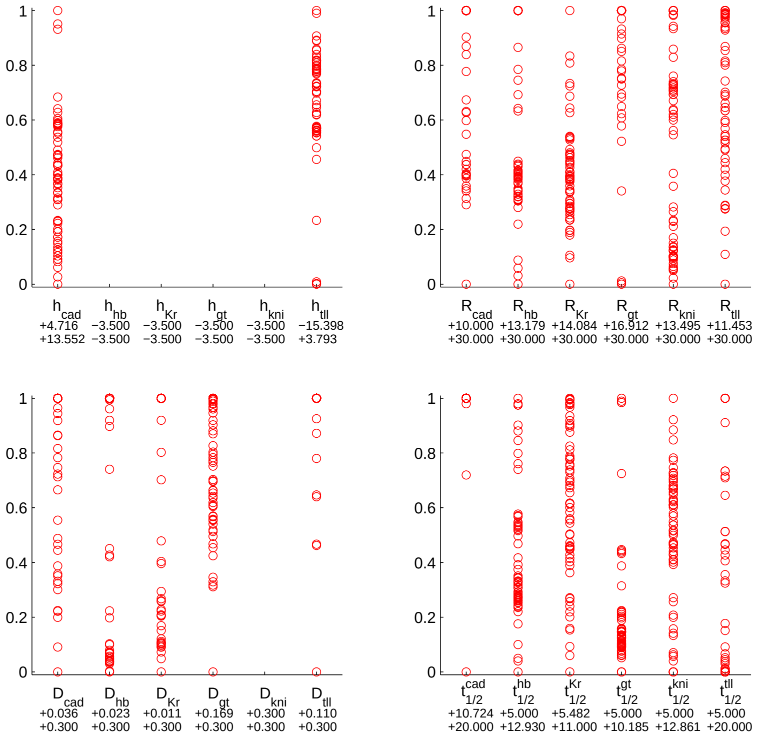

Figure 3.3: Scatter plots of parameters h, R, D and $t_{1/2} = ln(2)/\lambda$ (fixed case).

tory weights and maternal coefficients in the gap gene model. Dependent (green lines) and independent (red lines) confidence intervals are plotted along the vertical axis for all 60 parameter sets. Similar to full search case, small dependent confidence intervals in comparison with corresponding independent confidence intervals give us indication for presence of correlations between parameters. Therefore, we consider only independent confidence intervals in our assessment of the parameter determinability.

There is a quantitative improvement indicated by smaller confidence intervals for some regulatory weights and maternal coefficients in the fixed case in comparison with the results obtained in the full search case. In addition to eight regulatory weights which have small confidence intervals in the full search case, there are five regulatory weights, such as W^{cad}_{hb}, W^{hb}_{hb}, W^{cad}_{Kr}, W^{cad}_{gt}, and W^{cad}_{kni}, having the same property in the fixed case. The remaining weights have larger confidence intervals.

Based on the confidence intervals, we summarize the qualitative conclusions for the essential regulatory weights in the gap gene system.

- Cad activates gap genes hb, Kr, gt, and kni. Note that independent confidence intervals for this weights in all solutions entirely fall into positive part of the plane.
- Activation of hb, Kr, and gt by Bcd is confirmed in some solutions. However, there are also many solutions when the independent confidence intervals for these weights include zero. Therefore, one can make only the qualitative conclusion that Bcd does

not repress *hb*, *Kr*, and *gt*. Note that it is weaker conclusion than predicting the activation for these weights from Table 1 (paper).

- No conclusions can be made for regulation of *kni* by Bcd because the independent confidence intervals significantly extend to negative part of the plane.
- Autoactivation for gap genes *hb*, *Kr*, and *gt* is confirmed. Independent confidence intervals for these weights entirely fall into positive part of the plane with only a few exceptions in each case.
- Autoactivation of *kni* is less confirmative. Model only predicts that *kni* does not repress itself which is weaker conclusion than predicting the autoactivation for this weight from Table 1 (paper).
- Tll does not regulate *hb*.
- Tll does not activate gap gene *gt* which is weaker conclusion than predicting repression for this weight from Table 1 (paper).
- No conclusions can be made for regulation of Tll on *Kr* and *kni*.
- Mutually exclusive gap genes *gt* and *Kr* repress each other. Despite of the large sizes, the independent confidence intervals for these weights entirely fall into negative part of the plane in almost all parameter estimates.
- No conclusions can be made for regulation of *hb* on *kni* and *kni* on *hb* because their independent confidence intervals significantly extend to positive part of the plane;

The obtained qualitative conclusions have better agreement with the theory in comparison with the conclusions (B1)-(B4). However, they are still weaker than the conclusions made by considering only the values of parameter estimates.

We note that similar to full search case, promoter strengths R, diffusion coefficients D, and decay rates λ for all genes and promoter thresholds h for *cad* and *tll* have extremely large independent confidence intervals (see Figure 3.5) meaning that all these parameters are nondeterminable.

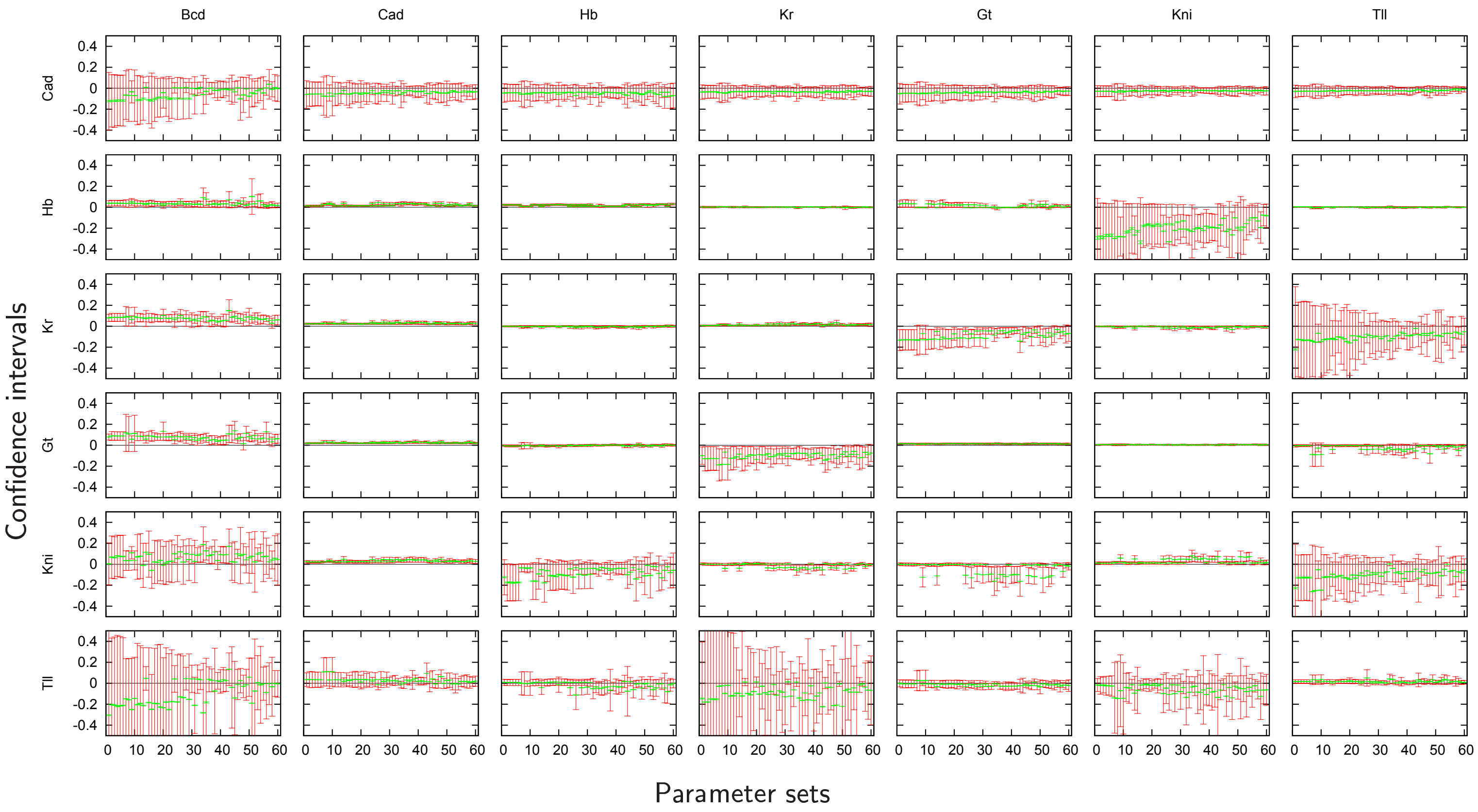

Figure 3.4: Confidence intervals for regulatory weights and maternal coefficients (fixed case).

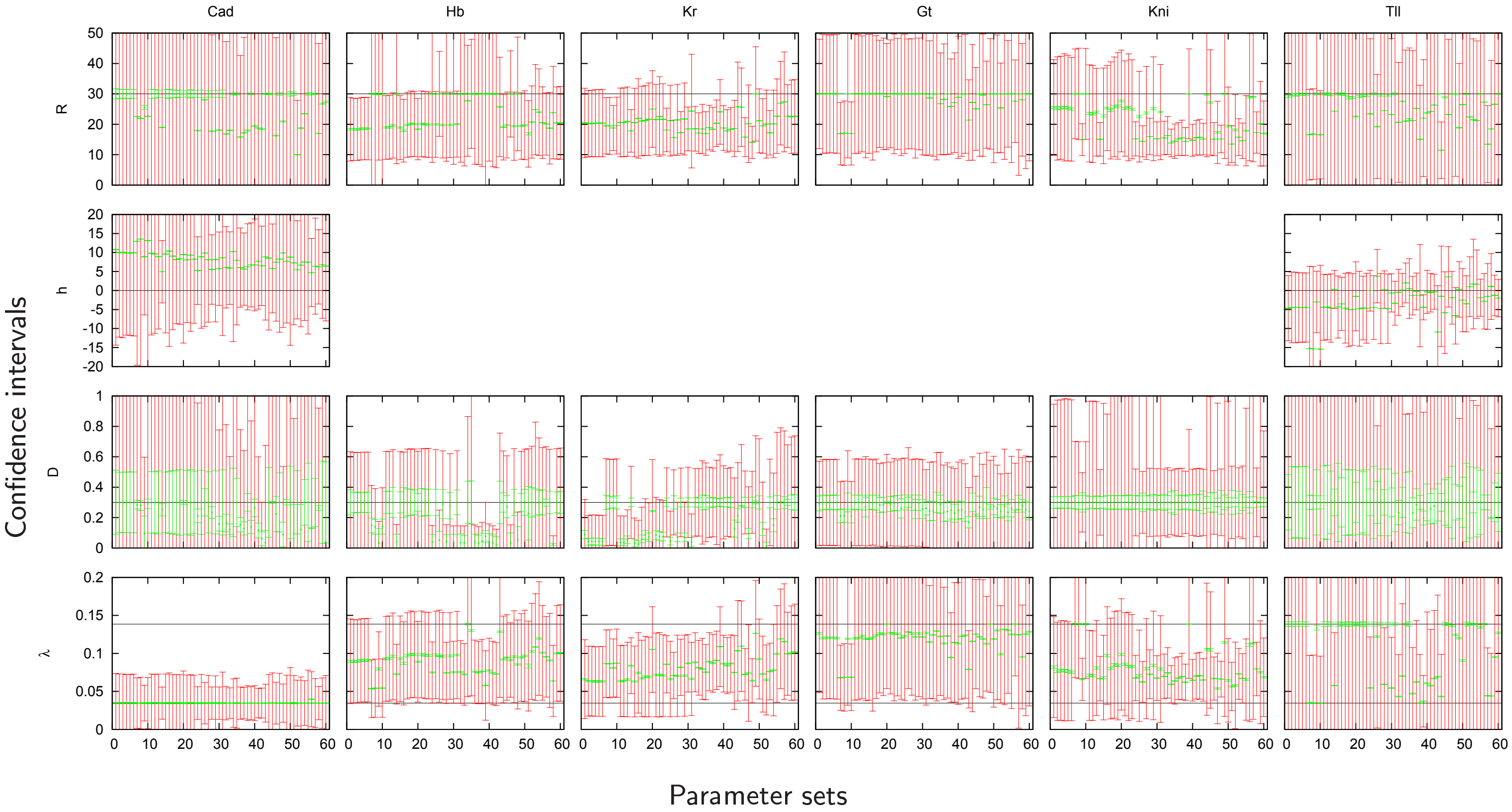

Figure 3.5: Confidence intervals for parameters h, R, D and λ (fixed case).

3.2 Correlations between parameters in the case of fixed promoter thresholds

By using formula (16), we find the correlation matrix for each parameter set in the fixed case. To detect the most significant correlations between parameters present in all correlation matrices, we calculate the mean correlation matrix shown in Figure 3.6. The obtained mean correlation matrix has a block diagonal structure. However, there is also a number of significant entries in off-diagonal blocks. In the absence of dominating correlations between regulatory parameters and thresholds h_a we can now identify biologically significant parameter correlations (see paper).

Similar to the full searcg case, the correlations corresponding to the most significant entries in the mean correlation matrix (with absolute values greater than 0.5) are statistically present in all individual correlation matrices because corresponding standard deviations are relatively small (less than 0.2).

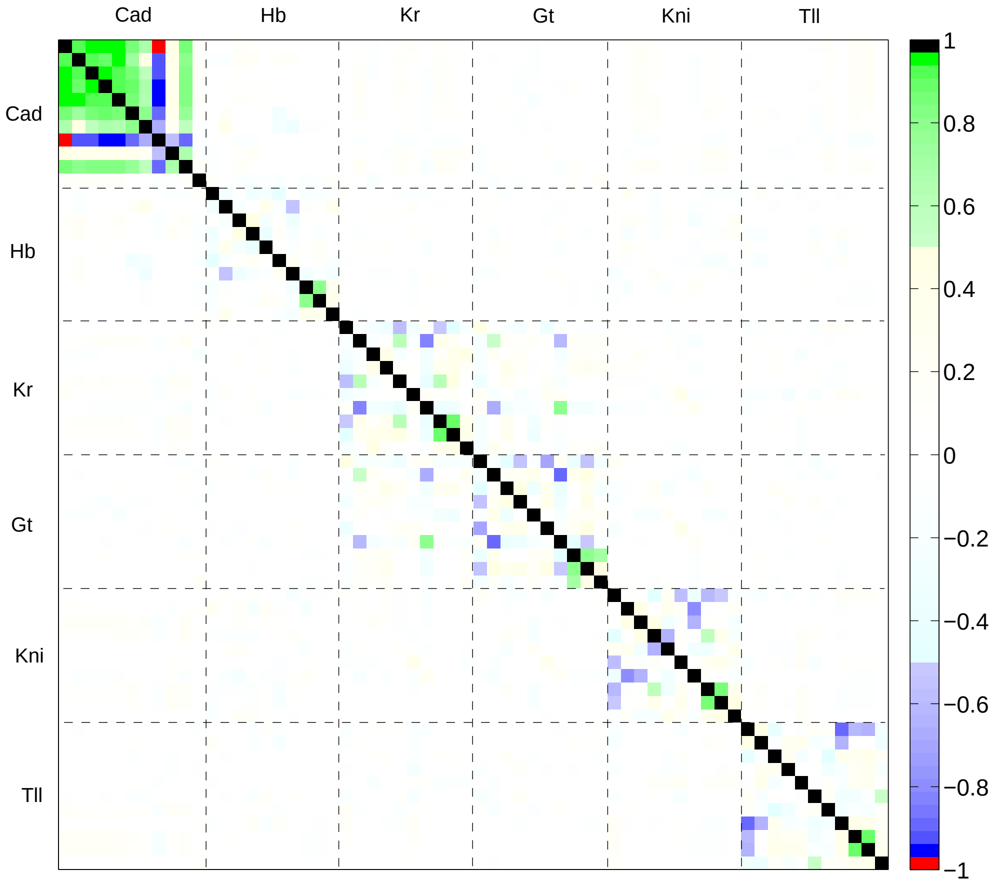

Figure 3.6: Mean correlation matrix (fixed case).

References

[1] B. W. Char, K. O. Gedded, G. H. Gonnet, B. L. Leong, M. B. Monagan, S. M. Watt (1991), *Maple V Library Reference manual*, Springer Verlag.

[2] Yves Fomekong Nanfack, Jaap A. Kaandorp, and Joke Blom (2007), *Efficient parameter estimation for spatio-temporal models of pattern formation: Case study of Drosophila melanogaster*, Bioinformatics 23, pp. 3356-3363.

[3] J. Jaeger, M. Blagov, D. Kosman, K. N. Kozlov, Manu, E. Myasnikova, S. Surkova, C. E. Vanario-Alonso, M. Samsonova, D. H. Sharp, and J. Reinitz (2004), *Dynamical analyses of regulatory interactions in the gap gene system of Drosophila melanogaster*, Genetics 167, pp. 1721-1737.

[4] J. Jaeger, S. Surkova, M. Blagov, H. Janssens, D. Kosman, K. N. Kozlov, Manu, E. Myasnikova, C. E. Vanario-Alonso, M. Samsonova, D. H. Sharp, and J. Reinitz (2004), *Dynamic control of positional information in the early Drosophila embryo*, Nature 430, pp. 368-371.

[5] E. Poustelnikova, A. Pisarev, M. Blagov, M. Samsonova, and J. Reinitz (2004). *A database for management of gene expression data in situ*, Bioinformatics 20, pp. 2212-2221. (http://flyex.ams.sunysb.edu/flyex)

[6] J. Jaeger and J. Reinitz (2006), *On the dynamic nature of positional information*, BioEssays 28, pp. 1102-1111.
